# Comparison of diagnostic accuracy for diabetes diagnosis: A systematic review and network meta-analysis

**DOI:** 10.3389/fmed.2023.1016381

**Published:** 2023-01-24

**Authors:** Khanh N. C. Duong, Chia Jie Tan, Sasivimol Rattanasiri, Ammarin Thakkinstian, Thunyarat Anothaisintawee, Nathorn Chaiyakunapruk

**Affiliations:** ^1^Department of Pharmacotherapy, College of Pharmacy, University of Utah, Salt Lake City, UT, United States; ^2^Department of Clinical Epidemiology and Biostatistics, Faculty of Medicine, Ramathibodi Hospital, Mahidol University, Bangkok, Thailand; ^3^Department of Family Medicine, Faculty of Medicine, Ramathibodi Hospital, Mahidol University, Bangkok, Thailand; ^4^Informatics, Decision-Enhancement, and Analytic Sciences (IDEAS) Center, Veterans Affairs Salt Lake City Health Care System, Salt Lake City, UT, United States

**Keywords:** systematic review, diabetic diagnosis, HbA1c, FPG, testing accuracy

## Abstract

**Aim:**

Fasting Plasma Glucose (FPG) and Hemoglobin A1c (HbA1c) are used as diagnostic tests for diagnosing diabetes mellitus, but it is unclear which test has the best diagnostic accuracy. This systematic review and network meta-analysis aimed to estimate the diagnostic accuracy of HbA1c ≥ 6.5%, FPG ≥ 126 mg/dl, and the combination of HbA1c ≥ 6.5% or FPG ≥ 126 mg/dl (HbA1c| FPG), compared with Oral Glucose Tolerance Test (OGTT) ≥ 200 mg/dl for diagnosis diabetes.

**Materials and methods:**

We performed a comprehensive search in PubMed, Embase, Cochrane Library, and Scopus from inception to September 24th, 2021. Inclusion criteria were any study design comparing HbA1c ≥ 6.5%, FPG ≥ 126 mg/dl, and HbA1c ≥ 6.5% or FPG ≥ 126 mg/dl with OGTT ≥ 200 mg/dl as the reference test. Data were independently extracted, risk of bias was assessed using QUADAS-2 by two reviewers. Network meta-analysis was done using a bivariate regression model using the Bayesian framework. The relative ranking of all tests was also assessed.

**Results:**

Out of 5,026 studies, 73 were included. The sensitivities of HbA1c, FPG, and HbA1c| FPG were 0.51 [95% Credible Interval (CrI): 0.43, 0.58], 0.49 (95% CrI: 0.43, 0.55), and 0.64 (95% CrI: 0.51, 0.75), while the specificities were 0.96 (95% CrI: 0.94, 0.97), 0.98 (95% CrI: 0.97, 0.98), and 0.95 (95% CrI: 0.88, 0.98), respectively. The corresponding positive likelihood ratios (LR) were 13.36 (95% CrI: 8.91, 20.72), 21.94 (95% CrI: 15.04, 31.88), and 11.78 (95% CrI: 5.48, 26.56). HbA1c| FPG is superior based on sensitivity, whereas FPG is ranked best based on specificity and positive LR.

**Conclusion:**

Our findings suggest that FPG ≥ 126 mg/dl should be recommended as the best diagnostic test for diabetes.

**Systematic review registration:**

https://www.crd.york.ac.uk/prospero/, identifier CRD42021282856.

## 1. Introduction

The American Diabetes Association (ADA) and World Health Organization (WHO) have established diagnostic criteria for diabetes based on one of three laboratory tests: fasting plasma glucose (FPG) > 7.0 mmol/L (126 mg/dl), 2-h plasma glucose > 11.1 mmol/L (200 mg/dl) in the oral glucose tolerance test (OGTT), and Hemoglobin A1c (HbA1c) > 6.5% (1, 2). FPG and OGTT directly measure glucose concentration in the blood plasma and have long been used in diagnosing diabetes (2). The former requires a sample to be taken after 8 h of fasting, while the latter involves an additional piece to be taken 2 h after consuming 75 g of anhydrous glucose dissolved in water. While the WHO criteria of OGTT is widely used as the gold standard, it has often been criticized for being time-consuming and inconvenient for patients, who must fast for 8 h prior to the test and wait for 2 h before a final blood sample can be drawn after glucose is taken (3).

HbA1c measures the glycosylation of the hemoglobin A1c chain and reflects plasma glucose levels in the past 2–3 months due to the relatively long lifespan of red blood cells (4). It was first established as the gold standard for monitoring glycemic control and guiding diabetes therapy (4). However, using HbA1c to diagnose diabetes was challenging due to the lack of standardized assay methods until recently (2). With the implementation of stringent quality assurance tests and international reference values, HbA1c was recommended by ADA and WHO for the diagnosis of diabetes in 2009 and 2011 (1, 2). Advantages of HbA1c compared to plasma glucose levels include less day-to-day variability, less susceptibility to short-term changes due to factors such as diet and stress, and increased convenience for patients as fasting is not necessary (3, 4). Given the increased convenience and relative ease of testing, FPG and HbA1c are potential alternatives to OGTT. However, some studies raised concerns about the diagnostic agreement between these tests (5, 6). The study revealed that HbA1c ≥ 6.5% was less likely to detect diabetes than FPG and OGTT (6) whereas another study showed better performance of HbA1c than FPG in estimating the prevalence of diabetes (5).

The previous meta-analyses conducted by Hoyer et al. in 2018 (7) and Kaur et al. in 2020 (8) pooled the sensitivity and specificity of FPG and HbA1c. However, it is still inconclusive which test has the best diagnostic accuracy. This study, therefore, aimed to quantify the diagnostic accuracy [i.e., sensitivity, specificity, positive likelihood ratio (LR+), negative likelihood ratio (LR–), and diagnostic odds ratio (DOR)] of HbA1c, FPG, and a combination of both methods relative to a standard OGTT for diabetic diagnosis. In addition, diagnostic performances between the two tests (i.e., HbA1c and FPG) were compared and ranked applying a network meta-analysis.

## 2. Materials and methods

This study was conducted following the PRISMA guideline for systematic review and meta-analysis and registered on the PROSPERO (Registration number: CRD42021282856).

### 2.1. Search strategy

We performed a comprehensive literature search using four electronic databases, PubMed, Embase, Cochrane Library, and Scopus, from inception to September 24th, 2021. The search strategy was based on the keywords search terms and synonyms for diabetes, HbA1c, FPG, OGTT, and diagnostic accuracy. Search strategies were modified according to specific electronic databases without language restriction, see Supplementary Table 1. The reference lists of selected studies were also explored to identify further relevant studies. Unpublished literature was not included in this review.

### 2.2. Study selection

The studies with any study designs were included if (1) they investigated HbA1c or FPG or the combination of both for diabetes diagnosis in adults, (2) the reference test was 75-g OGTT or 2-h post-load glucose through venous route (OGTT 2 h) ≥ 200 mg/dl (11.1 mmol/L), (3) the studies reported sufficient data for analysis [i.e., number of true positives (TP), false positives (FP), false negatives (FN), and true negatives (TN) or sensitivity and specificity of the tests of interest]. Studies on gestational diabetes were excluded. Article screening was conducted independently by two reviewers (KD and CT). Any disagreement was resolved by discussion, and if necessary, the opinion of a third reviewer (NC) was sought. Reference management and duplicates were removed in EndNote X9, and the screening process was conducted in Rayyan.

### 2.3. Index tests and reference standard tests

The index tests of interest were HbA1c ≥ 6.5% (48.0 mmol/L), FPG ≥ 7.0 mmol/L (or ≥ 126 mg/dl), and the combination of HbA1c ≥ 6.5% or FPG ≥ 126 mg/dl (HbA1c| FPG).

The outcome of interest was diabetes diagnosed by the standard OGTT 2h ≥ 11.1 mmol/L (or ≥ 200 mg/dl).

### 2.4. Data extraction and quality assessment

Data were independently extracted by two reviewers (KD and CT). Any disagreement was resolved by discussion with a third reviewer (NC). The extracted data included (1) article characteristics (author, year of publication), (2) study characteristics (country, study setting, study design, type of population), (3) index and reference tests (types, cut-offs for diabetic diagnosis, duration between index and reference tests), and (4) data for pooling (numbers of TP, FP, FN, and TN, or sensitivity and specificity).

Quality assessment was conducted independently by the same reviewers using the Quality Assessment of Diagnostic Accuracy Studies—2 (QUADAS-2) (9), considering the risk of bias and applicability. There are four domains in the QUADAS-2 tool, including patient selection, index test, reference test, and flow and timing. The risk of bias was assessed in all four domains, whereas concerns of applicability were applied for the first three domains.

### 2.5. Data synthesis and analyses

The diagnostic accuracy (i.e., sensitivity, specificity, LR+, LR–, DOR) of HbA1c, FPG, and combined HbA1c| FPG relative to OGTT were estimated for individual studies. These diagnostic performances were then directly pooled across studies using a bivariate logit equation (10).

The relative diagnostic performances among three index tests were then estimated using a bivariate random-effects network meta-analysis model proposed by Owen et al. (11). In particular, a Markov chain Monte Carlo (MCMC) methods with the Bayesian framework was used to account for heterogeneity, correlations between sensitivity and specificity data pairs. Non-informative prior distributions were specified for the test-specific accuracy parameters. The relative ranking of interest indexed was also estimated from this model. Technical details of this approach are documented in Supplementary Method.

All analyses were performed using STATA software, version 17.0 (StataCorp, College Station, TX, USA) and WinBUGS, version 1.4.3 software. The models were run for a burn-in of 10,000 iterations, followed by 50,000 iterations for parameters estimates.

## 3. Results

### 3.1. Study selection

**FIGURE 1:**
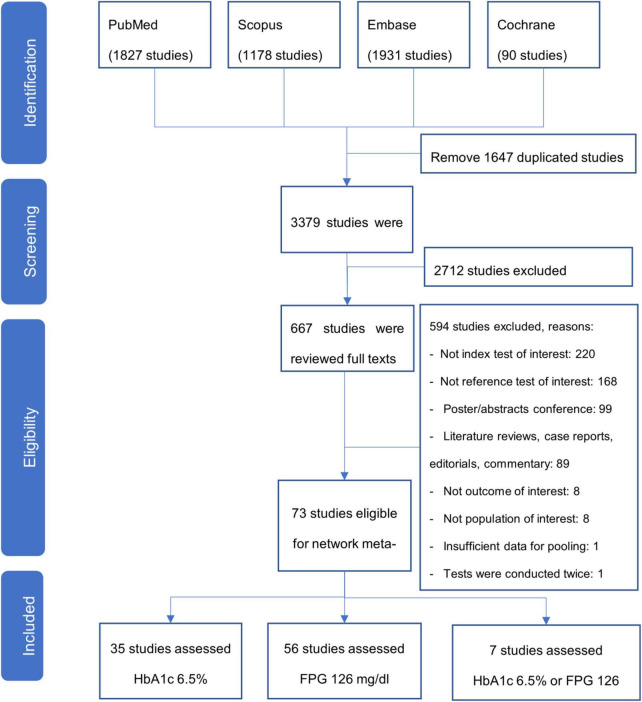
PRISMA flow chart of study selection.

A total of 5,026 studies were identified from database searches, of which 1,638 duplicates were removed, and the remaining records were screened based on the title and abstract. Of 676 full texts, 73 studies met the inclusion criteria and were included in the network meta-analysis. The overall workflow of the study selection is shown in the PRISMA flow diagram (Figure 1).

### 3.2. Study characteristics

Among 73 included studies involving 139,277 patients, 35, 56, and 7 studies assessed the performance of HbA1c, FPG, and the combined HbA1c| FPG, respectively. Forty-six studies (63%) and 27 studies (37%) recruited patients from clinical and community settings, respectively. Forty-one studies (56%) included the general population, whereas 32 studies (44%) included a high-risk population (e.g., individuals with the presence of one or more cardiometabolic risk factors including elevated blood pressure, dyslipidemia, overweight/obesity and family history for diabetes). Most of the studies (68 studies, 93%) were cross-sectional in nature, while the remaining five (7%) were cohort studies. Most of the studies (58 studies, 79.5%) reported that they performed the index tests and standard test on the same day whereas 13 studies (17.8%) did not mention it. The general characteristics of eligible studies are described in Supplementary Table 2.

The diagnostic accuracy (i.e., sensitivity, specificity, LR+, LR–, DOR) of three index tests relative to OGTT of individual studies are presented in Supplementary Tables 3–5, respectively. The paired forest plot of sensitivity and specificity along with data for TP, FP, FN, and TN of the included studies for each index test are depicted in Supplementary Figures 1–3.

### 3.3. Risk of bias assessment

Among 35 studies assessing HbA1c vs. OGTT, 34 (97.1%) and 32 studies (91.4%) were low risk in the index test and reference standard domains, respectively. Five (14.3%) and 14 studies (40%) had high or unclear bias in patient selection and flow and timing. All HbA1c studies demonstrated low concerns of applicability with regards to the index and reference tests whereas eight studies (22.9%) showed high applicability concerns in patient selection. This was due to inadequate reporting of the time interval between the index and the reference tests, the exclusion of subjects with invalid test results, or those lost to follow up.

For FPG vs. OGTT, 45 (80.4%), 53 (94.6%), 49 (87.5%), and 36 (64.3%) studies had low risk of bias in patient selection, index test, reference test, and flow and timing, respectively. All FPG studies had low concerns of applicability in the domains of index and reference tests while 14 studies (25%) showed high or unclear bias in the domain of patient selection. All studies investigating HbA1c ≥ 6.5% or FPG ≥ 126 mg/dl were evaluated to have low concerns of applicability in all domains and low risk of bias in patient selection. One study (14.3%) had unclear risk of bias with regards to the index test and reference standard, and three studies (42.9%) did not report the interval between the reference standard and index test. Specific details on the risk of bias assessment are presented in Supplementary Table 6.

### 3.4. Network meta-analysis of diagnostic tests

**FIGURE 2:**
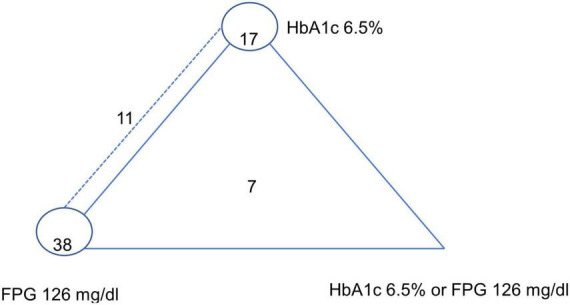
Network plot of HbA1c 6.5%, FPG 126 mg/dl, and HbA1c 6.5% or FPG 126 mg/dl (reference standard: OGTT-2 h 200 mg/dl). The dashed line represents HbA1c 6.5%–FPG 126 mg/dl paired-test studies, the closed triangle with solid line represents triple-test studies, and the closed circle refers to single test studies.[7 studies investigated all three index tests (i.e., HbA1c ≥ 6.5%, FPG ≥ 126 mg/dl, and HbA1c/FPG); 11 studies investigated two index tests HbA1c ≥ 6.5%, FPG ≥ 126 mg/dl; 17 studies investigated only HbA1c ≥ 6.5%, 38 studies investigated only FPG ≥ 126 mg/dl].

A network map of the 73 included studies is depicted in Figure 2. A total of 7 and 11 studies conducted multiple-test designs by investigate all three index tests (i.e., HbA1c ≥ 6.5%, FPG ≥ 126 mg/dl, and HbA1c/FPG), and two index-tests (HbA1c and FPG) whereas the remaining studies 38 and 17 studies were single-test designs by performing only one index test of FPG and HbA1c, respectively (single test design).

#### 3.4.1. HbA1C vs. OGTT

Using the bivariate random-effects network meta-analysis, the pooled sensitivity, specificity, LR+, LR–, and DOR along with their 95% Credible Interval (CrI) of HbA1c compared to OGTT were 0.51 (95% CrI: 0.43, 0.58), 0.96 (95% CrI: 0.94, 0.97), 13.36 (95% CrI: 8.91, 20.72), 0.51 (95% CrI: 0.44, 0.60), and 26.10 (95% CrI: 15.75, 44.07), respectively (Table 1).

**TABLE 1:** Performance of index tests versus OGTT.

Index vs. OGTT	Sensitivity (95% CrI)	Specificity (95% CrI)	LR+ (95% CrI)	LR– (95% CrI)	DOR (95% CrI)
HbA1c 6.5%	0.51 (0.43, 0.58)	0.96 (0.94, 0.97)	13.36 (8.91, 20.72)	0.51 (0.44, 0.60)	26.10 (15.75, 44.07)
FPG 126 mg/dl	0.49 (0.43, 0.55)	0.98 (0.97, 0.98)	21.94 (15.04, 31.88)	0.52 (0.46, 0.59)	41.86 (26.75, 64.62)
HbA1c 6.5% or FPG 126 mg/dl	0.64 (0.51, 0.75)	0.95 (0.88, 0.98)	11.78 (5.48, 26.56)	0.38 (0.26, 0.52)	31.50 (12.24, 83.44)

CrI, credible interval; DOR, diagnostic odds ratio; LR+, positive likelihood ratio; LR–, negative likelihood ratio.

#### 3.4.2. FPG vs. OGTT

With OGTT as standard test, the pooled accuracy performance of FPG (i.e., sensitivity, specificity, LR+, LR–, and DOR) were 0.49 (95% CrI: 0.43, 0.55), 0.98 (95% CrI: 0.97, 0.98), 21.94 (95% CrI: 15.04, 31.88), 0.52 (95% CrI: 0.46, 0.59), and 41.86 (95% CrI: 26.75, 64.62), respectively (Table 1).

#### 3.4.3. HbA1c|FPG vs. OGTT

When using the combination of HbA1c or FPG for diabetes diagnosis, the pool estimate for sensitivity was 0.64 (95% CrI: 0.51, 0.75), the specificity was 0.95 (95% CrI: 0.88, 0.98), the LR+ was 11.78 (95% CrI: 5.48, 26.56), the LR– was 0.38 (95% CrI: 0.26, 0.52), the LR– was 0.38 (95% CrI: 0.26, 0.52), and DOR was 31.50 (95% CrI: 12.24, 83.44) (Table 1).

#### 3.4.4. Fagan plots

Using OGTT-2 h with a cut-off of 200 mg/dl as the reference, 73 studies were used for pooling prevalence of diabetes. The pooled prevalence of diabetes across studies were 0.19 (95% CI = 0.17, 0.20; *I*^2^ = 99.16%) (see Supplementary Table 7). The Fangan Plots of three tests were depicted in Supplementary Figure 4. Given a pretest probability of diabetes of 19%, the post-test probability was increased to 76% if HbA1c was positive or reduced to 11% if HbA1c was negative (see Supplementary Figure 4A). With the same prevalence of diabetes (19%), a positive FPG would result in a post-test probability of 84% whereas a negative FPG would reduce the probability to 11% (see Supplementary Figure 4B). For HbA1c| FPG, given the prevalence of diabetes of 19%, if the test was positive, the probability of getting diabetes would increase to 73% and decreased to 8% if the test was negative (see Supplementary Figure 4C).

#### 3.4.5. Ranking

The relative rankings among the three tests are depicted in Table 2. In particular, in terms of relative ranking based on sensitivity, the combination of HbA1c ≥ 6.5% or FPG ≥ 126 mg/dl was in the first ranking. There appeared to be minimal difference between HbA1c ≥ 6.5% and FPG ≥ 126 mg/dl, the former test ranked the second place and the latter test ranked the third place. The highest specificity was demonstrated by FPG ≥ 126 mg/dl (95% CrI: 1, 2) while the second and last rank belonged to HbA1c 6.5, and HbA1c ≥ 6.5% or FPG ≥ 126 mg/dl, accordingly. The LR+ of FPG ≥ 126 mg/dl ranked the first place, followed by HbA1c ≥ 6.5% and HbA1c| FPG. The FPG test also had a highest LR–, followed by HbA1c and HbA1c| FPG. In terms of ranking based on DOR, FPG ≥ 126 mg/dl ranked the first place, followed by HbA1c ≥ 6.5% or FPG ≥ 126 mg/dl and HbA1c ≥ 6.5% (Table 2).

**TABLE 2:** The relative ranking of index tests.

Index vs. OGTT	Rank sensitivity (95% CrI)	*P* (best) sensitivity	Rank specificity (95% CrI)	*P* (best) specificity	Rank LR+ (95% CrI)	*P* (best) LR+	Rank LR– (95% CrI)	*P* (best) LR–	Rank DOR (95% CrI)	*P* (best) DOR
HbA1c 6.5%	2 (2, 3)	0.017	2 (2, 3)	0.019	2 (1, 3)	0.026	2 (1, 2)	0.394	3 (1, 3)	0.034
FPG 126 mg/dl	3 (2, 3)	0.006	1 (1, 2)	0.964	1 (1, 3)	0.905	1 (1, 1)	0.595	1 (1, 3)	0.689
HbA1c 6.5% or FPG 126 mg/dl	1 (1, 1)	0.977	3 (2, 3)	0.017	3 (1, 3)	0.069	3 (2, 3)	0.011	2 (1, 3)	0.277

CrI, credible interval; DOR, diagnostic odds ratio; LR+, positive likelihood ratio; LR–, negative likelihood ratio.

## 4. Discussion

This systematic review and network-meta-analysis included 73 studies with 139,277 patients, summarizing the evidence on diagnostic accuracy outcomes (sensitivity, specificity, DOR, LR+, and LR–) for three index tests, HbA1c ≥ 6.5%, FPG ≥ 126 mg/dl, and the combination of HbA1c ≥ 6.5% or FPG ≥ 126 mg/dl in diagnosing diabetes. The findings suggested that when using OGTT ≥ 200 mg/dl as the reference test, the diagnostic performance of FPG ≥ 126 mg/dl was the best in diagnosis of diabetes (i.e., highest LR+) among the three index tests of interest. However, the combination of HbA1c/FPG yielded the highest sensitivity, followed by HbA1c and FPG for rule-out purpose whereas FPG alone was the best for rule-in purpose, followed by HbA1c alone, and the combination thereof. In other words, for instance, out of 100 diabetic patients identified by OGTT as a reference, FPG could identify 49 patients, HbA1c and HbA1c| FPG could identify additional 2 and 15 patients (the sensitivity of FPG, HbA1c, and HbA1c| FPG were 0.49, 0.51, and 0.64, respectively). However, given the same context, HbA1c| FPG and HbA1c could be misclassified as diabetes in more 3 and 2 patients than FPG (the specificity of HbA1c| FPG, HbA1c, and FPG were 0.95, 0.96, and 0.98).

Our estimate of pooled sensitivity for HbA1c ≥ 6.5% (0.51) is very similar to the value reported in the meta-analysis by Kaur 2020 (0.50) (8). However, our summarized sensitivity is lower than that reported in Hoyer et al.’s meta-analysis (0.684) (7). On the contrary, our pooled specificity for HbA1c ≥ 6.5% (0.96) is lower than that reported by Kaur 2020 (0.97) (8) and in line with those in Hoyer, 2018 (0.96) (7). Regarding FPG ≥ 126 mg/dl, our pooled sensitivity and specificity (0.49 and 0.98, respectively) were lower than those in the previous meta-analysis (0.59 and 0.99, respectively) (8). Compared with the results in Hoyer’s study, our estimates were lower in sensitivity (0.69) and higher in specificity (0.96) (7). Hoyer, 2018’s study only included studies that conducted both test HbA1c and FPG to pool data while our studies included eligible studies which investigated at least one test of interest (12). Our study leveraged the availability of limited studies about diagnostic accuracy testing, increasing the sample size and increasing, and improving the precision of our estimates as a consequence. Regarding Kaur’s meta-analysis, a bivariate model was applied to pool data separately for each test (13). This method assumed that patients undergoing different index-tests within the same study are independent subgroups (14). Using Owen’s approach, our study took the interaction between effects of study and index tests into account, which could enhance the accuracy of our estimates (11). Notably, to the best of our knowledge, there were no studies that ranked the testing for diabetes diagnosis. Our network meta-analysis performed the relative rankings between the tests, therefore, answered the question of which test was considered the best for diagnosing diabetes.

There are several strengths of that are notable in our systematic review and network-meta-analysis. Firstly, we analyzed the pooled estimates of diagnostic accuracy of the index tests with the use of a bivariate network meta-analysis model, which considered the correlations between multiple sensitivity and specificity data pairs from the same study (11). Second, we estimated relative rankings of these interested index tests based on accuracy parameter accounted both sensitivity and specificity (i.e., LR+, DOR) incorporated uncertainty estimations.

Nevertheless, our present work has some limitations. Firstly, we selected studies with only one threshold for each index test considering the multiple thresholds, so we did not estimate the optimal threshold for these index tests. Given the increasing evidence on lower thresholds for diabetes screening (15, 16), further research is needed to investigate the accuracy of these tests and clinical meanings at the lower thresholds. Secondly, we did not explore the heterogeneity of pooled diagnostic accuracy and undertake sub-group analysis based on the potential variables, such as ethnicity, population or study setting.

OGTT, FPG, and HbA1c have all been recommended by the ADA and WHO as methods to diagnose diabetes, whereas FPG and HbA1c are more widely used due to their relative convenience compared to OGTT. Based on our analysis, FPG is more accurate than HbA1c in diabetes diagnosis and, therefore, should be adopted as the diagnostic test of choice for diabetes. Additionally, despite the convenience of HbA1c, this test is unavailable in many low-income settings due to the need for precision and standardization of analytic methods (17–19), our findings provide critical evidence to support the recommendation of FPG as the preferred diagnostic method for diabetes, especially in limited resources areas. However, more studies on health economic evaluations of these screening tests should be carried out in these settings, to give a comprehensive assessment and make a better decision on resource allocations. Moreover, our estimates also contributed to the more accurate estimates of the accuracy performance of these common tests in diagnosing diabetes, which could enhance future research on health technology assessments of these tests.

## Data availability statement

The data analyzed in this study is subject to the following licenses/restrictions: The datasets used and/or analyzed during the current study are available from the corresponding author on reasonable request. Requests to access these datasets should be directed to NC, nathorn.chaiyakunapruk@utah.edu.

## Author contributions

KD, CT, SR, AT, TA, and NC: conceptualization and writing – review and editing. KD, CT, and NC: study selection, data extraction and risk of bias assessment, and writing – original draft. KD, CT, and SR: formal analysis. KD, SR, AT, and NC: methodology. NC: supervision. All authors contributed to the article and approved the submitted version.

## References

[1] World Health Organization [WHO]. *Definition and diagnosis of diabetes mellitus and intermediate hyperglycaemia: report of a WHO/IDF consultation.* Geneva: World Health Organization (2006).

[2] American Diabetes Association. Diagnosis and classification of diabetes mellitus. *Diabetes Care.* (2011) 34 (Suppl. 1):S62–9. 10.2337/dc11-S062 21193628 PMC3006051

[3] Higgins T. HbA1c for screening and diagnosis of diabetes mellitus. *Endocrine.* (2013) 43:266–73. 10.1007/s12020-012-9768-y 22907625

[4] Ding L Xu Y Liu S Bi Y Xu Y. Hemoglobin A1c and diagnosis of diabetes. *J Diabetes.* (2018) 10:365–72. 29292842 10.1111/1753-0407.12640

[5] Jeon J Ko S Kwon H Kim N Kim J Kim C Prevalence of diabetes and prediabetes according to fasting plasma glucose and HbA1c. *Diabetes Metab J.* (2013) 37:349–57. 24199164 10.4093/dmj.2013.37.5.349 PMC3816136

[6] Karnchanasorn R Huang J Ou H Feng W Chuang L Chiu K Comparison of the current diagnostic criterion of HbA1c with fasting and 2-hour plasma glucose concentration. *J Diabetes Res.* (2016) 2016:6195494. 10.1155/2016/6195494 27597979 PMC4997021

[7] Hoyer A Rathmann W Kuss O. Utility of HbA(1c) and fasting plasma glucose for screening of Type 2 diabetes: a meta-analysis of full ROC curves. *Diabet Med.* (2018) 35:317–22. 10.1111/dme.13560 29230866

[8] Kaur G Lakshmi P Rastogi A Bhansali A Jain S Teerawattananon Y Diagnostic accuracy of tests for type 2 diabetes and prediabetes: a systematic review and meta-analysis. *PLoS One.* (2020) 15:e0242415. 10.1371/journal.pone.0242415 33216783 PMC7678987

[9] Whiting P Rutjes A Westwood M Mallett S Deeks J Reitsma J QUADAS-2: a revised tool for the quality assessment of diagnostic accuracy studies. *Ann Internal Med.* (2011) 155:529–36. 22007046 10.7326/0003-4819-155-8-201110180-00009

[10] Furuya-Kanamori L Kostoulas P Doi SAR. A new method for synthesizing test accuracy data outperformed the bivariate method. *J Clin Epidemiol.* (2021) 132:51–8. 10.1016/j.jclinepi.2020.12.015 33333166

[11] Owen R Cooper N Quinn T Lees R Sutton A. Network meta-analysis of diagnostic test accuracy studies identifies and ranks the optimal diagnostic tests and thresholds for health care policy and decision-making. *J Clin Epidemiol.* (2018) 99:64–74. 10.1016/j.jclinepi.2018.03.005 29548843

[12] Hoyer A Kuss O. Meta-analysis for the comparison of two diagnostic tests to a common gold standard: a generalized linear mixed model approach. *Stat Methods Med Res.* (2018) 27:1410–21. 10.1177/0962280216661587 27487844

[13] Leeflang M Deeks J Takwoingi Y Macaskill P. Cochrane diagnostic test accuracy reviews. *Syst Rev.* (2013) 2:82. 10.1186/2046-4053-2-82 24099098 PMC3851548

[14] Veroniki A Tsokani S Rücker G Mavridis D Takwoingi Y. Challenges in Comparative Meta-Analysis of the Accuracy of Multiple Diagnostic Tests. In: Evangelou E Veroniki A editors. *Meta-research: methods and protocols.* New York, NY: Springer (2022). p. 299–316. 10.1007/978-1-0716-1566-9_18 34550598

[15] Crowther C Samuel D McCowan L Edlin R Tran T McKinlay C. Lower versus higher glycemic criteria for diagnosis of gestational diabetes. *N Engl J Med.* (2022) 387:587–98. 36070709 10.1056/NEJMoa2204091

[16] Greene M. Drawing the line on glycemia in pregnancy. *N Engl J Med.* (2022) 387:652–4. 10.1056/NEJMe2208339 36070714

[17] Camargo M Passos L Mistro S Soares D Kochergin C de Carvalho V Improving Access to the Glycated Hemoglobin Test in Rural Communities With Point-of-Care Devices: an Application Study. *Front Med.* (2021) 8:734306. 10.3389/fmed.2021.734306 34881257 PMC8645789

[18] Klatman E Ogle G. Access to insulin delivery devices and glycated haemoglobin in lower-income countries. *World J Diabetes.* (2020) 11:358–69. 10.4239/wjd.v11.i8.358 32864048 PMC7438184

[19] Weykamp C. HbA1c: a review of analytical and clinical aspects. *Ann Lab Med.* (2013) 33:393–400. 10.3343/alm.2013.33.6.393 24205486 PMC3819436

