# Supplementary material for: Comparison of diagnostic accuracy for diabetes diagnosis: A systematic review and network meta-analysis
Source: Front Med (Lausanne). 2023 Jan 24;10:1016381. doi: 10.3389/fmed.2023.1016381 (PMC9902703; doi:10.3389/fmed.2023.1016381)
Supplement: Supplementary file 1 [file Data_Sheet_1.pdf]

Supplementary Material

Supplementary Method: WinBugs code for Network meta-analysis

```
model{

temp.var<- id[1]

# Bivariate model
      for(i in 1:nObs){                   #Loop through observations

      pos[i] <- tp[i] + fn[i]
      neg[i] <- fp[i] + tn[i]
    tp[i]~dbin(pi[i,1], pos[i])
      tn[i]~dbin(pi[i,2], neg[i])

      logit(pi[i,1]) <- mu[i,1]
      logit(pi[i,2]) <- mu[i,2]

# Model for linear predictor
      mu[i,1] <-  logitsens[test[i]] + study.re.sens[s[i]] +
test.re.sens[s[i],test[i]]
      mu[i,2] <-  logitspec[test[i]] + study.re.spec[s[i]] +
test.re.spec[s[i],test[i]]

                                                }

# Back transform on to sensitivity and specificity scale

      for(j in 1:ntest){                        # Loop through the number of
tests

            sens[j]<- exp(logitsens[j])/(1+exp(logitsens[j]))
            spec[j]<- exp(logitspec[j])/(1+exp(logitspec[j]))
            DOR[j]<- (sens[j]*spec[j])/((1-sens[j])*(1-spec[j]))
            LRpos[j]<- sens[j]/(1-spec[j])
            LRneg[j]<- (1-sens[j])/spec[j]
```

```
# Priors on the fixed test and threshold effects
      logitsens[j]~dnorm(0,0.001)
      logitspec[j]~dnorm(0,0.001)
                                                }
# Priors on the random study and test effects
      for(k in 1:ns){                           # Loop through the number of studies
      study.re.sens[k]~dnorm(0,taustudysens)
      study.re.spec[k]~dnorm(0,taustudyspec)

            for(l in 1:ntest){
             # Loop through the number of tests
                  test.re.sens[k,l]~dnorm(0,tautestsens)
                  test.re.spec[k,l]~dnorm(0,tautestspec)
                                                      }
                                          }

      taustudysens <- pow(SDstudysens,-2)
      SDstudysens ~ dunif(0,2)

      taustudyspec <- pow(SDstudyspec,-2)
      SDstudyspec ~ dunif(0,2)

      tautestsens <- pow(SDtestsens,-2)
      SDtestsens ~ dunif(0,2)

      tautestspec <- pow(SDtestspec,-2)
      SDtestspec ~ dunif(0,2)


# Calculate ranks and probability best
            for(l in 1:totaltest){
# Loop through total number of tests
            rksens[l]<-totaltest+1-rank(logitsens[],l)
            bestsens[l]<-equals(rksens[l],1)

            rkspec[l]<-totaltest+1-rank(logitspec[],l)
```

```
            bestspec[l]<-equals(rkspec[l],1)

            rkDOR[l]<-totaltest+1-rank(DOR[],l)
            bestDOR[l]<-equals(rkDOR[l],1)
            }
}
```

Study	True positive	False positive	False negative	True negative	Sensitivity (95% CI)	Specificity (95% CI)
Wiener 1998	52	0	126	223	0.29 (0.23, 0.36)	1.00 (0.98, 1.00)
Buck 2000	3	8	10	81	0.23 (0.05, 0.54)	0.91 (0.83, 0.96)
Tanaka 2001	87	14	91	674	0.49 (0.41, 0.56)	0.98 (0.97, 0.99)
Gianchadani 2011	1	3	2	84	0.33 (0.01, 0.91)	0.97 (0.90, 0.99)
Hanna 2011	3	2	38	155	0.07 (0.02, 0.20)	0.99 (0.95, 1.00)
lee 2011	28	7	63	1736	0.31 (0.22, 0.41)	1.00 (0.99, 1.00)
Adamu 2011	5	6	3	17	0.63 (0.24, 0.91)	0.74 (0.52, 0.90)
Valentine 2011	2	3	2	36	0.50 (0.07, 0.93)	0.92 (0.79, 0.98)
Yoon 2011	34	3	5	194	0.87 (0.73, 0.96)	0.98 (0.96, 1.00)
Zemlin 2011	67	17	80	655	0.46 (0.37, 0.54)	0.97 (0.96, 0.99)
Homko 2012	15	16	15	149	0.50 (0.31, 0.69)	0.90 (0.85, 0.94)
Marini 2012	61	54	70	834	0.47 (0.38, 0.55)	0.94 (0.92, 0.95)
Adamska 2012	17	10	20	394	0.46 (0.29, 0.63)	0.98 (0.95, 0.99)
Bianchi 2012	189	128	162	287	0.54 (0.48, 0.59)	0.69 (0.64, 0.74)
De Mulder 2012	11	0	27	71	0.29 (0.15, 0.46)	1.00 (0.95, 1.00)
Vehik 2012	68	15	190	1709	0.26 (0.21, 0.32)	0.99 (0.99, 1.00)
kumpatla 2013	50	58	34	637	0.60 (0.48, 0.70)	0.92 (0.89, 0.94)
lee 2013	878	192	672	2874	0.57 (0.54, 0.59)	0.94 (0.93, 0.95)
Alqahtani 2013	340	189	148	1137	0.70 (0.65, 0.74)	0.86 (0.84, 0.88)
Franco 2014	67	27	43	411	0.61 (0.51, 0.70)	0.94 (0.91, 0.96)
Karnchanasorn 2016	110	36	282	5336	0.28 (0.24, 0.33)	0.99 (0.99, 1.00)
Kim 2016	45	7	37	147	0.55 (0.43, 0.66)	0.95 (0.91, 0.98)
Aviles-Santa 2016	186	311	496	14514	0.27 (0.24, 0.31)	0.98 (0.98, 0.98)
Camacho 2016	1	1	6	167	0.14 (0.00, 0.58)	0.99 (0.97, 1.00)
Herath 2017	32	38	9	175	0.78 (0.62, 0.89)	0.82 (0.76, 0.87)
Joung 2018	196	24	76	219	0.72 (0.66, 0.77)	0.90 (0.86, 0.94)
Lopez 2018	49	7	191	866	0.20 (0.16, 0.26)	0.99 (0.98, 1.00)
Nam 2018	60	10	8	311	0.88 (0.78, 0.95)	0.97 (0.94, 0.98)
Prakashchandra 2018	102	92	52	830	0.66 (0.58, 0.74)	0.90 (0.88, 0.92)
Coelho 2018	0	0	13	207	0.00 (0.00, 0.25)	1.00 (0.98, 1.00)
Aamir 2019	354	79	64	532	0.85 (0.81, 0.88)	0.87 (0.84, 0.90)
Thewjitcharoen 2019	32	25	69	386	0.32 (0.23, 0.42)	0.94 (0.91, 0.96)
Basit 2020	353	446	460	5577	0.43 (0.40, 0.47)	0.93 (0.92, 0.93)
Cetin 2020	17	33	17	134	0.50 (0.32, 0.68)	0.80 (0.73, 0.86)
Tucker 2020	186	54	327	6845	0.36 (0.32, 0.41)	0.99 (0.99, 0.99)
Overall					0.51 (0.43, 0.58)	0.96 (0.94, 0.97)

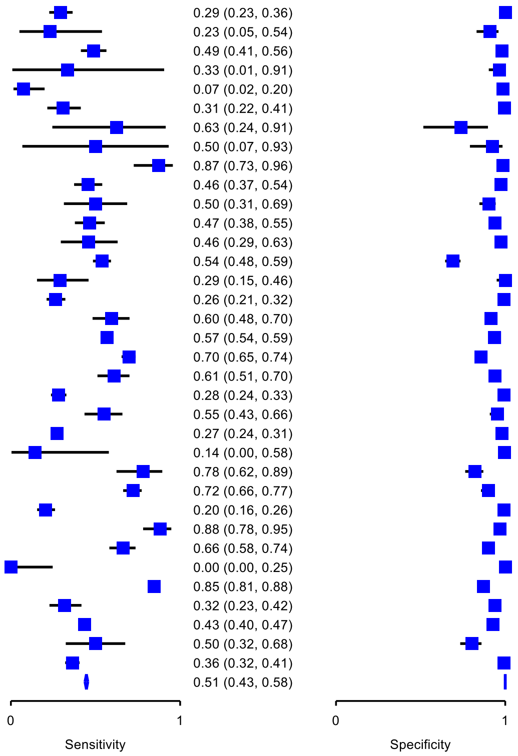

Supplementary Figure 1: Forest plot of sensitivity and specificity of HbA1c 6.5% for diabetic diagnosis (Note: The overall summary of sensitivity and specificity is presented with 95% credible interval)

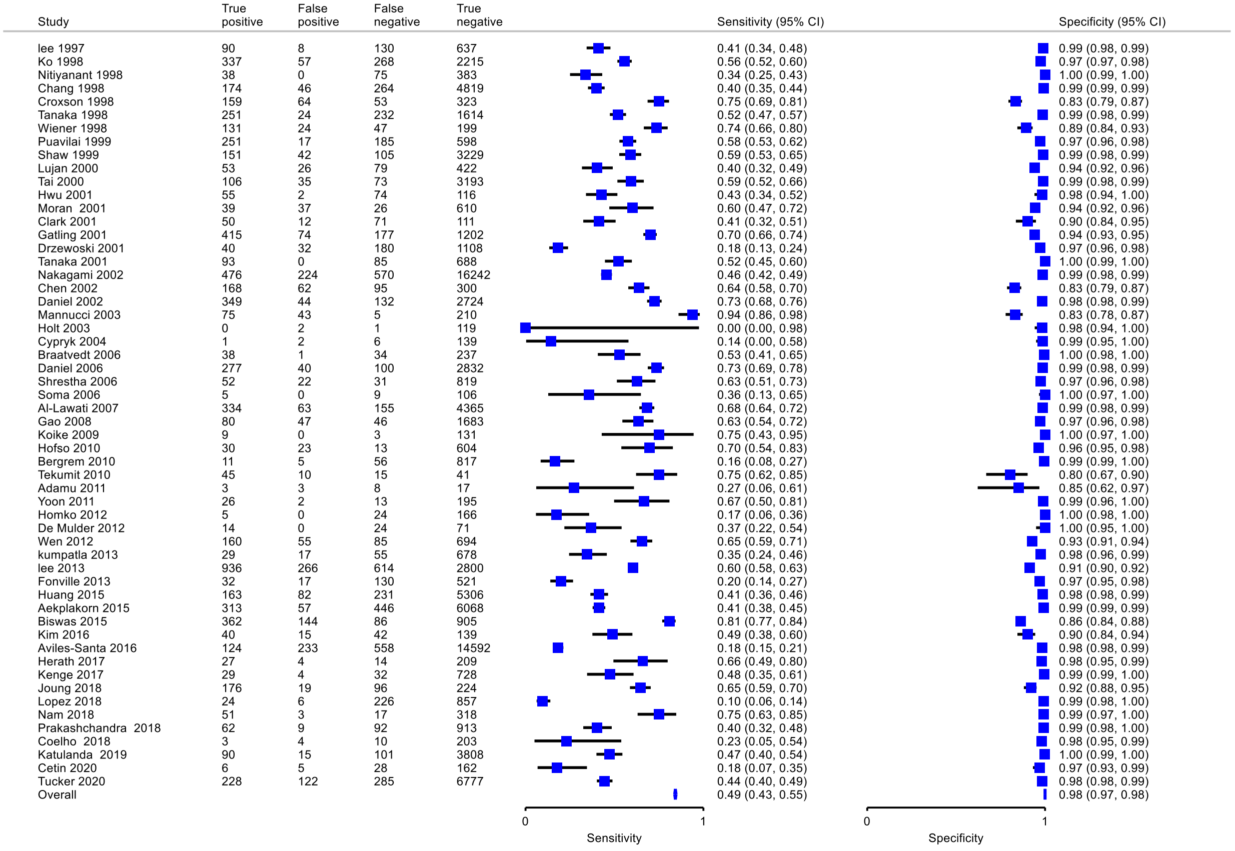

Study	True positive	False positive	False negative	True negative	Sensitivity (95% CI)	Specificity (95% CI)
lee 1997	90	8	130	637	0.41 (0.34, 0.48)	0.99 (0.98, 0.99)
Ko 1998	337	57	268	2215	0.56 (0.52, 0.60)	0.97 (0.97, 0.98)
Nitiyanant 1998	38	0	75	383	0.34 (0.25, 0.43)	1.00 (0.99, 1.00)
Chang 1998	174	46	264	4819	0.40 (0.35, 0.44)	0.99 (0.99, 0.99)
Croxson 1998	159	64	53	323	0.75 (0.69, 0.81)	0.83 (0.79, 0.87)
Tanaka 1998	251	24	232	1614	0.52 (0.47, 0.57)	0.99 (0.98, 0.99)
Wiener 1998	131	24	47	199	0.74 (0.66, 0.80)	0.89 (0.84, 0.93)
Puavilai 1999	251	17	185	598	0.58 (0.53, 0.62)	0.97 (0.96, 0.98)
Shaw 1999	151	42	105	3229	0.59 (0.53, 0.65)	0.99 (0.98, 0.99)
Lujan 2000	53	26	79	422	0.40 (0.32, 0.49)	0.94 (0.92, 0.96)
Tai 2000	106	35	73	3193	0.59 (0.52, 0.66)	0.99 (0.98, 0.99)
Hwu 2001	55	2	74	116	0.43 (0.34, 0.52)	0.98 (0.94, 1.00)
Moran 2001	39	37	26	610	0.60 (0.47, 0.72)	0.94 (0.92, 0.96)
Clark 2001	50	12	71	111	0.41 (0.32, 0.51)	0.90 (0.84, 0.95)
Gatling 2001	415	74	177	1202	0.70 (0.66, 0.74)	0.94 (0.93, 0.95)
Drzewoski 2001	40	32	180	1108	0.18 (0.13, 0.24)	0.97 (0.96, 0.98)
Tanaka 2001	93	0	85	688	0.52 (0.45, 0.60)	1.00 (0.99, 1.00)
Nakagami 2002	476	224	570	16242	0.46 (0.42, 0.49)	0.99 (0.98, 0.99)
Chen 2002	168	62	95	300	0.64 (0.58, 0.70)	0.83 (0.79, 0.87)
Daniel 2002	349	44	132	2724	0.73 (0.68, 0.76)	0.98 (0.98, 0.99)
Mannucci 2003	75	43	5	210	0.94 (0.86, 0.98)	0.83 (0.78, 0.87)
Holt 2003	0	2	1	119	0.00 (0.00, 0.98)	0.98 (0.94, 1.00)
Cypryk 2004	1	2	6	139	0.14 (0.00, 0.58)	0.99 (0.95, 1.00)
Braatvedt 2006	38	1	34	237	0.53 (0.41, 0.65)	1.00 (0.98, 1.00)
Daniel 2006	277	40	100	2832	0.73 (0.69, 0.78)	0.99 (0.98, 0.99)
Shrestha 2006	52	22	31	819	0.63 (0.51, 0.73)	0.97 (0.96, 0.98)
Soma 2006	5	0	9	106	0.36 (0.13, 0.65)	1.00 (0.97, 1.00)
Al-Lawati 2007	334	63	155	4365	0.68 (0.64, 0.72)	0.99 (0.98, 0.99)
Gao 2008	80	47	46	1683	0.63 (0.54, 0.72)	0.97 (0.96, 0.98)
Koike 2009	9	0	3	131	0.75 (0.43, 0.95)	1.00 (0.97, 1.00)
Hofso 2010	30	23	13	604	0.70 (0.54, 0.83)	0.96 (0.95, 0.98)
Bergrem 2010	11	5	56	817	0.16 (0.08, 0.27)	0.99 (0.99, 1.00)
Tekumit 2010	45	10	15	41	0.75 (0.62, 0.85)	0.80 (0.67, 0.90)
Adamu 2011	3	3	8	17	0.27 (0.06, 0.61)	0.85 (0.62, 0.97)
Yoon 2011	26	2	13	195	0.67 (0.50, 0.81)	0.99 (0.96, 1.00)
Homko 2012	5	0	24	166	0.17 (0.06, 0.36)	1.00 (0.98, 1.00)
De Mulder 2012	14	0	24	71	0.37 (0.22, 0.54)	1.00 (0.95, 1.00)
Wen 2012	160	55	85	694	0.65 (0.59, 0.71)	0.93 (0.91, 0.94)
kumpatla 2013	29	17	55	678	0.35 (0.24, 0.46)	0.98 (0.96, 0.99)
lee 2013	936	266	614	2800	0.60 (0.58, 0.63)	0.91 (0.90, 0.92)
Fonville 2013	32	17	130	521	0.20 (0.14, 0.27)	0.97 (0.95, 0.98)
Huang 2015	163	82	231	5306	0.41 (0.36, 0.46)	0.98 (0.98, 0.99)
Aekplakorn 2015	313	57	446	6068	0.41 (0.38, 0.45)	0.99 (0.99, 0.99)
Biswas 2015	362	144	86	905	0.81 (0.77, 0.84)	0.86 (0.84, 0.88)
Kim 2016	40	15	42	139	0.49 (0.38, 0.60)	0.90 (0.84, 0.94)
Aviles-Santa 2016	124	233	558	14592	0.18 (0.15, 0.21)	0.98 (0.98, 0.99)
Herath 2017	27	4	14	209	0.66 (0.49, 0.80)	0.98 (0.95, 0.99)
Kenge 2017	29	4	32	728	0.48 (0.35, 0.61)	0.99 (0.99, 1.00)
Joung 2018	176	19	96	224	0.65 (0.59, 0.70)	0.92 (0.88, 0.95)
Lopez 2018	24	6	226	857	0.10 (0.06, 0.14)	0.99 (0.98, 1.00)
Nam 2018	51	3	17	318	0.75 (0.63, 0.85)	0.99 (0.97, 1.00)
Prakashchandra 2018	62	9	92	913	0.40 (0.32, 0.48)	0.99 (0.98, 1.00)
Coelho 2018	3	4	10	203	0.23 (0.05, 0.54)	0.98 (0.95, 0.99)
Katulanda 2019	90	15	101	3808	0.47 (0.40, 0.54)	1.00 (0.99, 1.00)
Cetin 2020	6	5	28	162	0.18 (0.07, 0.35)	0.97 (0.93, 0.99)
Tucker 2020	228	122	285	6777	0.44 (0.40, 0.49)	0.98 (0.98, 0.99)
Overall					0.49 (0.43, 0.55)	0.98 (0.97, 0.98)

Supplementary Figure 2: Forest plot of sensitivity and specificity of FPG 126 mg/dl for diabetic diagnosis (Note: The overall summary of sensitivity and specificity is presented with 95% credible interval)

Study	True positive	False positive	False negative	True negative	Sensitivity (95% CI)	Specificity (95% CI)
Tanaka 2001	110	14	68	674	0.62 (0.54, 0.69)	0.98 (0.97, 0.99)
lee 2013	1151	434	399	2632	0.74 (0.72, 0.76)	0.86 (0.85, 0.87)
Aviles-Santa 2016	217	342	465	14483	0.32 (0.28, 0.35)	0.98 (0.97, 0.98)
Herath 2017	34	38	7	175	0.83 (0.68, 0.93)	0.82 (0.76, 0.87)
Joung 2018	213	37	59	206	0.78 (0.73, 0.83)	0.85 (0.80, 0.89)
Lopez 2018	62	9	188	854	0.25 (0.20, 0.31)	0.99 (0.98, 1.00)
Nam 2018	62	11	6	310	0.91 (0.82, 0.97)	0.97 (0.94, 0.98)
Overall					0.64 (0.51, 0.75)	0.95 (0.88, 0.98)

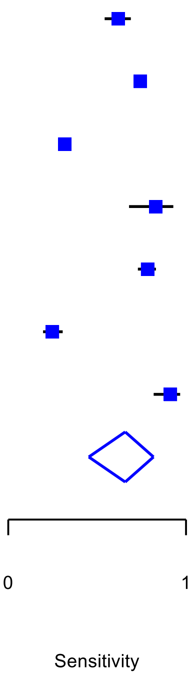

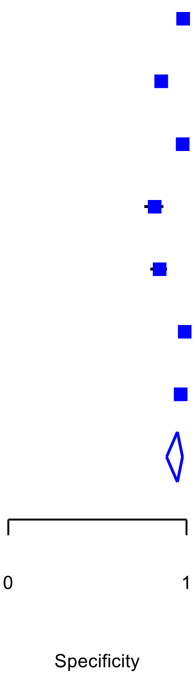

Supplementary Figure 3: Forest plot of sensitivity and specificity of HbA1c 6.5% or FPG 126 mg/dl for diabetic diagnosis (Note: The overall summary of sensitivity and specificity is presented with 95% credible interval)

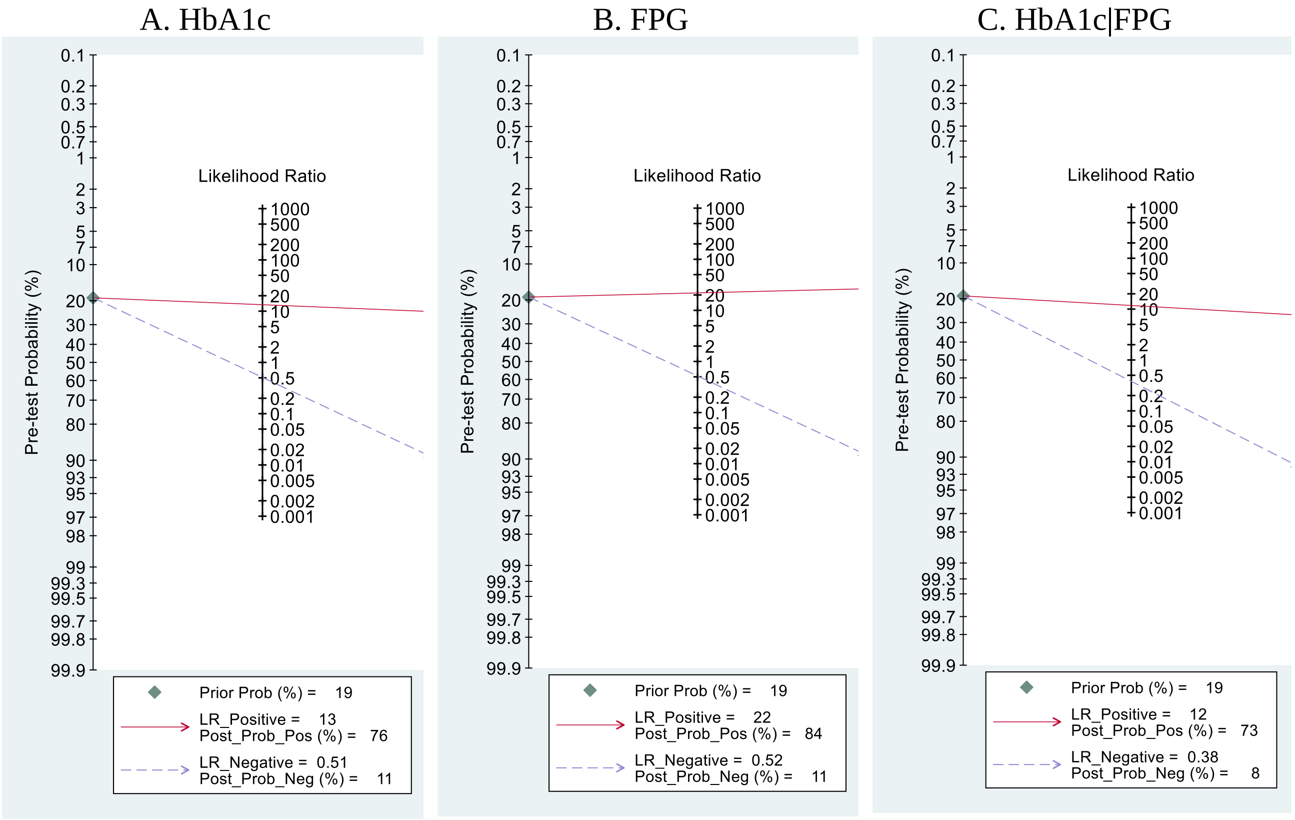

Supplementary Figure 4: Fagan's plots of (A): HbA1c, (B): FPG, and (C): HbA1c|FPG

Supplementary Table 1: Search strategy

**Medline database**	
#1	"diabetes mellitus"[MeSH Terms]
#2	diabetes[Title/Abstract]
#3	#1 OR #2
#4	"Fasting plasma glucose"[Title/Abstract]
#5	"Fasting Blood Glucose"[Title/Abstract]
#6	FPG[Title/Abstract]
#7	FBG[Title/Abstract]
#8	#4 OR #5 OR #6 OR #7
#9	HbA1c[Title/Abstract]
#10	A1c[Title/Abstract]
#11	#9 OR #10
#12	#8 OR #11
#13	ogtt
#14	"2 hour plasma glucose"[Title/Abstract]
#15	"2 h plasma glucose"[Title/Abstract]
#16	"2 hr plasma glucose"[Title/Abstract]
#17	"2h plasma glucose"[Title/Abstract]
#18	#14 OR #15 OR #16 OR #17
#19	sensitivity and specificity[MeSH Terms]
#20	sensitivity[Title/Abstract]
#21	specificity[Title/Abstract]
#22	"diagnostic performance"[Title/Abstract]
#23	"diagnostic accuracy"[Title/Abstract]
#24	#19 OR #20 OR #21 OR #22 OR #23
#25	#3 AND #12 AND #18 AND #24
**Scopus database**	
#1	TITLE-ABS-KEY (diabetes)
#2	TITLE-ABS-KEY ("Fasting plasma glucose")
#3	TITLE-ABS-KEY ("Fasting blood glucose")
#4	TITLE-ABS-KEY (fpg)
#5	TITLE-ABS-KEY (fbg)
#6	#2 OR #3 OR #4 OR #5
#7	TITLE-ABS-KEY (hba1c)
#8	TITLE-ABS-KEY (a1c)
#9	#7 OR #8
#10	#6 OR #9
#11	TITLE-ABS-KEY (ogtt)

#12	TITLE-ABS-KEY ("2 hour plasma glucose")
#13	TITLE-ABS-KEY ("2 h plasma glucose")
#14	TITLE-ABS-KEY ("2h plasma glucose")
#15	#11 OR #12 OR #13 OR #14
#16	TITLE-ABS-KEY ("sensitivity")
#17	TITLE-ABS-KEY ("specificity")
#18	TITLE-ABS-KEY ("diagnostic performance")
#19	TITLE-ABS-KEY ("diagnostic accuracy")
#20	#16 OR #17 OR #18 OR #19
#21	#1 AND #10 AND #15 AND #20
**Embase database**	
#1	'diabetes mellitus'/exp
#2	'diabetes':ti,ab
#3	#1 OR #2
#4	'fasting plasma glucose'/exp
#5	'fasting plasma glucose':ti,ab
#6	'fasting blood glucose':ab,ti
#7	'fbg':ab,ti
#8	'fpg':ab,ti
#9	#4 OR #5 OR #6 OR #7 OR #8
#10	'hemoglobin a1c':ab,ti
#11	'hba1c':ab,ti
#12	'a1c':ab,ti
#13	#10 OR #11 OR #12
#14	#9 OR #13
#15	'oral glucose tolerance test':ab,ti
#16	'2 hour plasma glucose':ab,t
#17	'2 h plasma glucose':ab,ti
#18	'2 hr plasma glucose':ab,ti
#19	'2h plasma glucose':ab,ti
#20	'ogtt':ab,ti
#21	#15 OR #16 OR #17 OR #18 OR #19 OR #20
#22	'sensitivity':ab,ti
#23	'specificity':ab,ti
#24	'diagnostic accuracy':ab,ti
#25	'diagnostic performance':ab,ti
#26	#22 OR #23 OR #24 OR #25
#27	#3 AND #14 AND #21 AND #26
**Cochrane Library**	
#1	MeSH descriptor: [Diabetes Mellitus] explore all trees

#2	("Fasting blood glucose"):ti,ab,kw
#3	("Fasting plasma glucose"):ti,ab,kw
#4	(FPG):ti,ab,kw
#5	(FBG):ti,ab,kw
#6	#2 OR #3 OR #4 OR #5
#7	HbA1c:ti,ab,kw
#8	A1c:ti,ab,kw
#9	#7 OR #8
#10	(OGTT):ti,ab,kw
#11	("2 hour plasma glucose"):ti,ab,kw
#12	("2 h plasma glucose"):ti,ab,kw
#13	("2 hr plasma glucose"):ti,ab,kw
#14	("2h plasma glucose"):ti,ab,kw
#15	#10 OR #11 OR #12 OR #13 OR #14
#16	('sensitivity'):ti,ab,kw
#17	('specificity'):ti,ab,kw
#18	('diagnostic accuracy'):ti,ab,kw
#19	('diagnostic performance'):ti,ab,kw
#20	#16 OR #17 OR #18 OR #19
#21	#6 OR #9
#22	#1 AND #15 AND #20 AND #21

Supplementary Table 2: Key characteristics of the included studies

Author	Year	Country	Study setting	Study design	Type of population	Blood Glucose Test	Time interval between index test and OGTT
Lee	1997	Singapore	Clinical setting	Cross-sectional	General population	FPG	Same day
Ko	1998	Hong Kong	Clinical setting	Cross-sectional	High-risk population	FPG	Same day
Nitiyanant	1998	Thailand	Clinical setting	Cross-sectional	High-risk population	FPG	Same day
Chang	1998	Taiwan	Community	Cross-sectional	General population	FPG	Same day
Croxson	1998	UK	Clinical setting	Cross-sectional	General population	FPG	Not mentioned
Tanaka	1998	Japan	Clinical setting	Cross-sectional	High-risk population	FPG	Same day
Wiener	1998	UK	Clinical setting	Cross-sectional	General population	HbA1c, FPG	Same day
Puavilai	1999	Thailand	Clinical setting	Cross-sectional	High-risk population	FPG	Same day
Shaw	1999	Mauritius	Community	Cohort	General population	FPG	Not mentioned
Lujan	2000	Spain	Clinical setting	Cross-sectional	High-risk population	FPG	Same day
Buck	2000	Germany	Clinical setting	Cross-sectional	General population	HbA1c	Same day
Tai	2000	Singapore	Community	Cross-sectional	General population	FPG	Same day
Hwu	2001	Taiwan	Clinical setting	Cross-sectional	High-risk population	FPG	Same day
Moran	2001	Mexico	Community	Cross-sectional	General population	FPG	Same day
Clark	2001	USA	Community	Cohort	High-risk population	FPG	Same day
Gatling	2001	UK	Clinical setting	Cross-sectional	General population	FPG	Not mentioned
Drzewoski	2001	Poland	Clinical setting	Cross-sectional	High-risk population	FPG	Same day

Author	Year	Country	Study setting	Study design	Type of population	Blood Glucose Test	Time interval between index test and OGTT
Tanaka	2001	Japan	Clinical setting	Cross-sectional	High-risk population	HbA1c, FPG, HbA1c|FPG	Same day
Nakagami	2002	Multiple countries	Clinical setting	Cross-sectional	General population	FPG	Not mentioned
Chen	2002	Singapore	Clinical setting	Cross-sectional	High-risk population	FPG	Same day
Daniel	2002	Australia	Community	Cross-sectional	High-risk population	FPG	Same day
Mannucci	2003	Italy	Community	cross-sectional	General population	FPG	Same day
Holt	2003	US	Clinical setting	Cross-sectional	High-risk population	FPG	Same day
Cypryk	2004	Poland	Clinical setting	Cross-sectional	High-risk population	FPG	Not mentioned
Braatvedt	2006	New Zealand	Clinical setting	Cross-sectional	General population	FPG	Same day
Daniel	2006	Australia	Community	Cross-sectional	General population	FPG	Same day
Shrestha	2006	Nepal	Community	Cross-sectional	General population	FPG	Same day
Soma	2006	South Africa	Clinical setting	Cross-sectional	High-risk population	FPG	Same day
Al-Lawati	2007	Oman	Community	Cross-sectional	General population	FPG	Same day
Gao	2008	China	Community	Cross-sectional	General population	FPG	Same day
Koike	2009	Japan	Clinical setting	Cross-sectional	General population	FPG	Not mentioned
Hofso	2010	Norway	Clinical setting	Cross-sectional	High-risk population	FPG	Not mentioned
Bergrem	2010	Norway	Clinical setting	Cross-sectional	High-risk population	FPG	Same day
Tekumit	2010	Turkey	Clinical setting	Cross-sectional	High-risk population	FPG	Same day
Gianchadani	2011	US	Clinical setting	Cross-sectional	High-risk population	HbA1c	Same day

Author	Year	Country	Study setting	Study design	Type of population	Blood Glucose Test	Time interval between index test and OGTT
Hanna	2011	UK	Clinical setting	Cross-sectional	High-risk population	HbA1c	Not mentioned
Lee	2011	US	Community	Cross-sectional	General population	HbA1c	Not mentioned
Adamu	2011	Nigeria	Clinical setting	Cross-sectional	High-risk population	HbA1c, FPG	Same day
Valentine	2011	Australia	Clinical setting	Cross-sectional	General population	HbA1c	Same day
Zemlin	2011	South Africa	Community	Cross-sectional	General population	HbA1c	Same day
Homko	2012	America	Community	Cross-sectional	High-risk population	HbA1c, FPG	Same day
Marini	2012	Italy	Community	Cross-sectional	High-risk population	HbA1c	Same day
Adamska	2012	Poland	Clinical setting	Cross-sectional	General population	HbA1c	Same day
Bianchi	2012	Italy	Community	Cohort	High-risk population	HbA1c	Not mentioned
De Mulder	2012	Netherlands	Clinical setting	Cross-sectional	High-risk population	HbA1c, FPG	Same day
Vehik	2012	Multiple countries	Clinical setting	Cohort	General population	HbA1c	With in 90 days
Wen	2012	China	Clinical setting	Cross-sectional	High-risk population	FPG	Same day
Kumpatla	2013	India	Clinical setting	Cross-sectional	General population	HbA1c, FPG	Same day
Lee	2013	Korea	Clinical setting	Cross-sectional	General population	HbA1c, FPG, HbA1c|FPG	Not mentioned
Alqahtani	2013	Saudi Arabia	Clinical setting	Cross-sectional	General population	HbA1c	Not mentioned
Fonville	2013	Netherlands	Clinical setting	Cross-sectional	High-risk population	FPG	Same day
Franco	2014	Brazil	Community	Cross-sectional	General population	HbA1c	Not mentioned
Huang	2015	US	Community	Cross-sectional	General population	FPG	Same day

Author	Year	Country	Study setting	Study design	Type of population	Blood Glucose Test	Time interval between index test and OGTT
Aekplakorn	2015	Thailand	Clinical setting	Cross-sectional	High-risk population	FPG	Same day
Biswas	2015	India	Clinical setting	Cross-sectional	General population	FPG	Not mentioned
Karnchanasorn	2016	US	Community	Cross-sectional	General population	HbA1c	Same day
Kim	2016	Korea	Clinical setting	Cross-sectional	High-risk population	HbA1c, FPG	Same day
Aviles-Santa	2016	USA	Community	Cross-sectional	General population	HbA1c, FPG, HbA1c/ FPG	Not mentioned
Camacho	2016	USA	Community	Cross-sectional	High-risk population	HbA1c	Same day
Herath	2017	Sri Lanka	Community	Cross-sectional	General population	HbA1c, FPG, HbA1c|FPG	Same day
Kengne	2017	South Africa	Clinical setting	Cross-sectional	General population	FPG	Not mentioned
Yoon	2018	South Korea	Clinical setting	Cross-sectional	High-risk population	HbA1c, FPG	Same day
Joung	2018	Korea	Clinical setting	Cross-sectional	High-risk population	HbA1c, FPG, HbA1c|FPG	Same day
Lopez	2018	Colombia	Community	Cross-sectional	General population	HbA1c, FPG, HbA1c|FPG	Same day
Nam	2018	Korea	Clinical setting	Cross-sectional	General population	HbA1c, FPG, HbA1c|FPG	Same day
Prakaschadra	2018	South Africa	Clinical setting	Cross-sectional	General population	HbA1c, FPG	Not mentioned
Coelho	2018	Portugal	Clinical setting	Cross-sectional	General population	HbA1c, FPG	Same day
Katulanda	2019	Sri Lanka	Community	Cross-sectional	General population	FPG	Same day
Aamir	2019	Pakistan	Community	Cross-sectional	General population	HbA1c	Within 7 days
Thewjitcharoen	2019	Thailand	Clinical setting	Cross-sectional	High-risk population	HbA1c	Same day

Author	Year	Country	Study setting	Study design	Type of population	Blood Glucose Test	Time interval between index test and OGTT
Basit	2020	Pakistan	Community	Cohort	General population	HbA1c	Same day
Cetin	2020	Turkey	Clinical setting	Cross-sectional	General population	HbA1c, FPG	Not mentioned
Tucker	2020	USA	Community	Cross-sectional	General population	HbA1c, FPG	Same day

Supplementary Table 3: Diagnostic performance of included studies assessed HbA1c 6.5% for diabetic diagnosis

Author	Year	TP	FP	FN	TN	Sensitivity (95%CI)	Specificity (95%CI)	LR+ (95%CI)	LR- (95%CI)	DOR (95%CI)
Wiener	1998	52	0	126	223	0.29 (0.23, 0.36)	1 (0.98, 1)	131.4 (8.17, 2113.94)	0.71 (0.64, 0.78)	185.51 (11.35, 3031.1)
Buck	2000	3	8	10	81	0.23 (0.05, 0.54)	0.91 (0.83, 0.96)	2.57 (0.78, 8.46)	0.85 (0.62, 1.15)	3.04 (0.69, 13.35)
Tanaka	2001	87	14	91	674	0.49 (0.41, 0.56)	0.98 (0.97, 0.99)	24.02 (14, 41.21)	0.52 (0.45, 0.6)	46.03 (25.12, 84.32)
Adamu	2011	5	6	3	17	0.63 (0.24, 0.91)	0.74 (0.52, 0.9)	2.4 (1, 5.73)	0.51 (0.2, 1.28)	4.72 (0.86, 26.04)
Gianchadani	2011	1	3	2	84	0.33 (0.01, 0.91)	0.97 (0.9, 0.99)	9.67 (1.38, 67.86)	0.69 (0.31, 1.54)	14 (0.98, 200.64)
Hanna	2011	3	2	38	155	0.07 (0.02, 0.2)	0.99 (0.95, 1)	5.74 (0.99, 33.25)	0.94 (0.86, 1.02)	6.12 (0.99, 37.92)
Valentine	2011	2	3	2	36	0.5 (0.07, 0.93)	0.92 (0.79, 0.98)	6.5 (1.5, 28.09)	0.54 (0.2, 1.45)	12 (1.22, 118.1)
Yoon	2011	34	3	5	194	0.87 (0.73, 0.96)	0.98 (0.96, 1)	57.25 (18.5, 177.11)	0.13 (0.06, 0.3)	439.73 (100.4, 1926.01)
Zemlin	2011	67	17	80	655	0.46 (0.37, 0.54)	0.97 (0.96, 0.99)	18.02 (10.91, 29.75)	0.56 (0.48, 0.65)	32.27 (18.05, 57.67)
lee	2011	28	7	63	1736	0.31 (0.22, 0.41)	1 (0.99, 1)	76.62 (34.39, 170.68)	0.7 (0.61, 0.8)	110.22 (46.38, 261.92)
Adamska	2012	17	10	20	394	0.46 (0.29, 0.63)	0.98 (0.95, 0.99)	18.56 (9.17, 37.56)	0.55 (0.41, 0.75)	33.49 (13.6, 82.46)
Bianchi	2012	189	128	162	287	0.54 (0.48, 0.59)	0.69 (0.64, 0.74)	1.75 (1.47, 2.08)	0.67 (0.59, 0.76)	2.62 (1.95, 3.52)
De Mulder	2012	11	0	27	71	0.29 (0.15, 0.46)	1 (0.95, 1)	42.46 (2.57, 701.44)	0.71 (0.58, 0.87)	59.8 (3.41, 1049.88)
Homko	2012	15	16	15	149	0.5 (0.31, 0.69)	0.9 (0.85, 0.94)	5.16 (2.87, 9.28)	0.55 (0.39, 0.79)	9.31 (3.85, 22.5)
Marini	2012	61	54	70	834	0.47 (0.38, 0.55)	0.94 (0.92, 0.95)	7.66 (5.58, 10.51)	0.57 (0.48, 0.67)	13.46 (8.67, 20.9)
Vehik	2012	68	15	190	1709	0.26 (0.21, 0.32)	0.99 (0.99, 1)	30.29 (17.59, 52.17)	0.74 (0.69, 0.8)	40.78 (22.86, 72.74)
Alqahtani	2013	340	189	148	1137	0.7 (0.65, 0.74)	0.86 (0.84, 0.88)	4.89 (4.23, 5.65)	0.35 (0.31, 0.41)	13.82 (10.8, 17.69)
Kumpatla	2013	50	58	34	637	0.6 (0.48, 0.7)	0.92 (0.89, 0.94)	7.13 (5.27, 9.66)	0.44 (0.34, 0.57)	16.15 (9.68, 26.95)
Lee	2013	878	192	672	2874	0.57 (0.54, 0.59)	0.94 (0.93, 0.95)	9.05 (7.83, 10.44)	0.46 (0.44, 0.49)	19.56 (16.38, 23.35)
Franco	2014	67	27	43	411	0.61 (0.51, 0.7)	0.94 (0.91, 0.96)	9.88 (6.66, 14.67)	0.42 (0.33, 0.53)	23.72 (13.74, 40.95)
Aviles-Santa	2016	186	311	496	14514	0.27 (0.24, 0.31)	0.98 (0.98, 0.98)	13 (11.03, 15.33)	0.74 (0.71, 0.78)	17.5 (14.29, 21.43)

Author	Year	TP	FP	FN	TN	Sensitivity (95%CI)	Specificity (95%CI)	LR+ (95%CI)	LR- (95%CI)	DOR (95%CI)
Camacho	2016	1	1	6	167	0.14 (0, 0.58)	0.99 (0.97, 1)	24 (1.67, 345.43)	0.86 (0.64, 1.17)	27.83 (1.55, 500.33)
Karnchanasorn	2016	110	36	282	5336	0.28 (0.24, 0.33)	0.99 (0.99, 1)	41.87 (29.15, 60.15)	0.72 (0.68, 0.77)	57.82 (38.95, 85.82)
Kim	2016	45	7	37	147	0.55 (0.43, 0.66)	0.95 (0.91, 0.98)	12.07 (5.7, 25.56)	0.47 (0.37, 0.6)	25.54 (10.66, 61.22)
Herath	2017	32	38	9	175	0.78 (0.62, 0.89)	0.82 (0.76, 0.87)	4.37 (3.14, 6.09)	0.27 (0.15, 0.48)	16.37 (7.22, 37.12)
Coelho	2018	0	0	13	207	0 (0, 0.25)	1 (0.98, 1)	14.86 (0.31, 721.22)	0.97 (0.87, 1.07)	15.37 (0.29, 805.06)
Joung	2018	196	24	76	219	0.72 (0.66, 0.77)	0.9 (0.86, 0.94)	7.3 (4.95, 10.74)	0.31 (0.26, 0.38)	23.53 (14.31, 38.71)
Lopez	2018	49	7	191	866	0.2 (0.16, 0.26)	0.99 (0.98, 1)	25.46 (11.68, 55.49)	0.8 (0.75, 0.86)	31.74 (14.16, 71.15)
Nam	2018	60	10	8	311	0.88 (0.78, 0.95)	0.97 (0.94, 0.98)	28.32 (15.29, 52.45)	0.12 (0.06, 0.23)	233.25 (88.43, 615.25)
Prakashchandra	2018	102	92	52	830	0.66 (0.58, 0.74)	0.9 (0.88, 0.92)	6.64 (5.3, 8.31)	0.38 (0.3, 0.47)	17.7 (11.89, 26.33)
Aamir	2019	354	79	64	532	0.85 (0.81, 0.88)	0.87 (0.84, 0.9)	6.55 (5.31, 8.08)	0.18 (0.14, 0.22)	37.25 (26.09, 53.18)
Thewjitcharoen	2019	32	25	69	386	0.32 (0.23, 0.42)	0.94 (0.91, 0.96)	5.21 (3.24, 8.38)	0.73 (0.64, 0.83)	7.16 (4, 12.82)
Basit	2020	353	446	460	5577	0.43 (0.4, 0.47)	0.93 (0.92, 0.93)	5.86 (5.21, 6.6)	0.61 (0.58, 0.65)	9.6 (8.1, 11.36)
Cetin	2020	17	33	17	134	0.5 (0.32, 0.68)	0.8 (0.73, 0.86)	2.53 (1.61, 3.99)	0.62 (0.44, 0.88)	4.06 (1.88, 8.79)
Tucker	2020	186	54	327	6845	0.36 (0.32, 0.41)	0.99 (0.99, 0.99)	46.32 (34.68, 61.87)	0.64 (0.6, 0.69)	72.1 (52.22, 99.56)

Supplementary Table 4: Diagnostic performance of included studies assessed FPG 126mg/dl for diabetic diagnosis

Author	Year	TP	FP	FN	TN	Sensitivity (95%CI)	Specificity (95%CI)	LR+ (95%CI)	LR- (95%CI)	DOR (95%CI)
Lee	1997	90	8	130	637	0.41 (0.34, 0.48)	0.99 (0.98, 0.99)	32.98 (16.27, 66.87)	0.6 (0.54, 0.67)	55.13 (26.11, 116.39)
Chang	1998	174	46	264	4819	0.4 (0.35, 0.44)	0.99 (0.99, 0.99)	42.01 (30.82, 57.28)	0.61 (0.56, 0.66)	69.05 (48.77, 97.76)
Croxson	1998	159	64	53	323	0.75 (0.69, 0.81)	0.83 (0.79, 0.87)	4.54 (3.58, 5.75)	0.3 (0.24, 0.38)	15.14 (10.04, 22.83)
Ko	1998	337	57	268	2215	0.56 (0.52, 0.6)	0.97 (0.97, 0.98)	22.2 (17.02, 28.97)	0.45 (0.42, 0.5)	48.86 (35.91, 66.49)
Nitiyanant	1998	38	0	75	383	0.34 (0.25, 0.43)	1 (0.99, 1)	259.37 (16.06, 4189.01)	0.66 (0.58, 0.76)	391.12 (23.77, 6436.28)
Tanaka	1998	251	24	232	1614	0.52 (0.47, 0.57)	0.99 (0.98, 0.99)	35.47 (23.63, 53.25)	0.49 (0.44, 0.53)	72.76 (46.82, 113.06)
Wiener	1998	131	24	47	199	0.74 (0.66, 0.8)	0.89 (0.84, 0.93)	6.84 (4.64, 10.08)	0.3 (0.23, 0.38)	23.11 (13.48, 39.62)
Puavilai	1999	251	17	185	598	0.58 (0.53, 0.62)	0.97 (0.96, 0.98)	20.83 (12.94, 33.51)	0.44 (0.39, 0.49)	47.73 (28.43, 80.13)
Shaw	1999	151	42	105	3229	0.59 (0.53, 0.65)	0.99 (0.98, 0.99)	45.94 (33.45, 63.1)	0.42 (0.36, 0.48)	110.56 (74.61, 163.84)
Lujan	2000	53	26	79	422	0.4 (0.32, 0.49)	0.94 (0.92, 0.96)	6.92 (4.51, 10.61)	0.64 (0.55, 0.73)	10.89 (6.43, 18.45)
Tai	2000	106	35	73	3193	0.59 (0.52, 0.66)	0.99 (0.98, 0.99)	54.62 (38.44, 77.6)	0.41 (0.35, 0.49)	132.47 (84.72, 207.13)
Clark	2001	50	12	71	111	0.41 (0.32, 0.51)	0.9 (0.84, 0.95)	4.24 (2.38, 7.55)	0.65 (0.55, 0.76)	6.51 (3.24, 13.08)
Drzewoski	2001	40	32	180	1108	0.18 (0.13, 0.24)	0.97 (0.96, 0.98)	6.48 (4.16, 10.08)	0.84 (0.79, 0.9)	7.69 (4.71, 12.57)
Gatling	2001	415	74	177	1202	0.7 (0.66, 0.74)	0.94 (0.93, 0.95)	12.09 (9.63, 15.17)	0.32 (0.28, 0.36)	38.08 (28.4, 51.07)
Hwu	2001	55	2	74	116	0.43 (0.34, 0.52)	0.98 (0.94, 1)	25.16 (6.27, 100.86)	0.58 (0.5, 0.68)	43.11 (10.21, 182.08)
Moran	2001	39	37	26	610	0.6 (0.47, 0.72)	0.94 (0.92, 0.96)	10.49 (7.24, 15.2)	0.42 (0.31, 0.57)	24.73 (13.61, 44.92)
Tanaka	2001	93	0	85	688	0.52 (0.45, 0.6)	1 (0.99, 1)	719.8 (44.91, 11537.52)	0.48 (0.41, 0.56)	1505.84 (92.65, 24475.79)
Chen	2002	168	62	95	300	0.64 (0.58, 0.7)	0.83 (0.79, 0.87)	3.73 (2.92, 4.76)	0.44 (0.37, 0.52)	8.56 (5.9, 12.41)
Daniel	2002	349	44	132	2724	0.73 (0.68, 0.76)	0.98 (0.98, 0.99)	45.65 (33.87, 61.51)	0.28 (0.24, 0.32)	163.68 (114.32, 234.36)
Nakagami	2002	476	224	570	16242	0.46 (0.42, 0.49)	0.99 (0.98, 0.99)	33.45 (28.91, 38.71)	0.55 (0.52, 0.58)	60.55 (50.61, 72.45)

Author	Year	TP	FP	FN	TN	Sensitivity (95%CI)	Specificity (95%CI)	LR+ (95%CI)	LR- (95%CI)	DOR (95%CI)
Holt	2003	0	2	1	119	0 (0, 0.98)	0.98 (0.94, 1)	12.2 (0.82, 180.78)	0.77 (0.34, 1.71)	15.93 (0.51, 495.43)
Mannucci	2003	75	43	5	210	0.94 (0.86, 0.98)	0.83 (0.78, 0.87)	5.52 (4.18, 7.28)	0.08 (0.03, 0.18)	73.26 (27.97, 191.88)
Cypryk	2004	1	2	6	139	0.14 (0, 0.58)	0.99 (0.95, 1)	10.07 (1.03, 98.21)	0.87 (0.64, 1.18)	11.58 (0.92, 146.26)
Braatvedt	2006	38	1	34	237	0.53 (0.41, 0.65)	1 (0.98, 1)	125.61 (17.55, 898.95)	0.47 (0.37, 0.61)	264.88 (35.21, 1992.57)
Daniel	2006	277	40	100	2832	0.73 (0.69, 0.78)	0.99 (0.98, 0.99)	52.75 (38.55, 72.19)	0.27 (0.23, 0.32)	196.12 (133.2, 288.76)
Shrestha	2006	52	22	31	819	0.63 (0.51, 0.73)	0.97 (0.96, 0.98)	23.95 (15.35, 37.36)	0.38 (0.29, 0.51)	62.45 (33.79, 115.4)
Soma	2006	5	0	9	106	0.36 (0.13, 0.65)	1 (0.97, 1)	78.47 (4.57, 1348.72)	0.64 (0.43, 0.94)	123.32 (6.33, 2404.16)
Al-Lawati	2007	334	63	155	4365	0.68 (0.64, 0.72)	0.99 (0.98, 0.99)	48.01 (37.29, 61.8)	0.32 (0.28, 0.37)	149.3 (109.14, 204.23)
Gao	2008	80	47	46	1683	0.63 (0.54, 0.72)	0.97 (0.96, 0.98)	23.37 (17.11, 31.91)	0.38 (0.3, 0.47)	62.28 (39.15, 99.07)
Koike	2009	9	0	3	131	0.75 (0.43, 0.95)	1 (0.97, 1)	192.92 (11.89, 3129.11)	0.27 (0.11, 0.66)	713.86 (34.3, 14856.68)
Bergrem	2010	11	5	56	817	0.16 (0.08, 0.27)	0.99 (0.99, 1)	26.99 (9.66, 75.41)	0.84 (0.76, 0.94)	32.1 (10.78, 95.58)
Hofso	2010	30	23	13	604	0.7 (0.54, 0.83)	0.96 (0.95, 0.98)	19.02 (12.17, 29.73)	0.31 (0.2, 0.49)	60.6 (27.99, 131.23)
Tekumit	2010	45	10	15	41	0.75 (0.62, 0.85)	0.8 (0.67, 0.9)	3.83 (2.15, 6.79)	0.31 (0.2, 0.49)	12.3 (4.98, 30.41)
Adamu	2011	3	3	8	17	0.27 (0.06, 0.61)	0.85 (0.62, 0.97)	1.82 (0.44, 7.53)	0.86 (0.57, 1.28)	2.13 (0.35, 12.95)
Yoon	2011	26	2	13	195	0.67 (0.5, 0.81)	0.99 (0.96, 1)	65.67 (16.25, 265.39)	0.34 (0.22, 0.53)	195 (41.64, 913.19)
De Mulder	2012	14	0	24	71	0.37 (0.22, 0.54)	1 (0.95, 1)	53.54 (3.28, 873.57)	0.63 (0.5, 0.81)	84.63 (4.86, 1472.41)
Homko	2012	5	0	24	166	0.17 (0.06, 0.36)	1 (0.98, 1)	61.23 (3.48, 1078.8)	0.82 (0.69, 0.97)	74.76 (4.01, 1394.41)
Wen	2012	160	55	85	694	0.65 (0.59, 0.71)	0.93 (0.91, 0.94)	8.89 (6.79, 11.65)	0.37 (0.31, 0.45)	23.75 (16.24, 34.74)
Fonville	2013	32	17	130	521	0.2 (0.14, 0.27)	0.97 (0.95, 0.98)	6.25 (3.57, 10.96)	0.83 (0.77, 0.9)	7.54 (4.06, 14.01)
kumpatla	2013	29	17	55	678	0.35 (0.24, 0.46)	0.98 (0.96, 0.99)	14.11 (8.11, 24.57)	0.67 (0.57, 0.78)	21.03 (10.88, 40.64)
lee	2013	936	266	614	2800	0.6 (0.58, 0.63)	0.91 (0.9, 0.92)	6.96 (6.16, 7.86)	0.43 (0.41, 0.46)	16.05 (13.65, 18.86)
Aekplakorn	2015	313	57	446	6068	0.41 (0.38, 0.45)	0.99 (0.99, 0.99)	44.31 (33.76, 58.16)	0.59 (0.56, 0.63)	74.71 (55.45, 100.67)

Author	Year	TP	FP	FN	TN	Sensitivity (95%CI)	Specificity (95%CI)	LR+ (95%CI)	LR- (95%CI)	DOR (95%CI)
Biswas	2015	362	144	86	905	0.81 (0.77, 0.84)	0.86 (0.84, 0.88)	5.89 (5.02, 6.9)	0.22 (0.18, 0.27)	26.45 (19.72, 35.48)
Huang	2015	163	82	231	5306	0.41 (0.36, 0.46)	0.98 (0.98, 0.99)	27.18 (21.28, 34.73)	0.6 (0.55, 0.65)	45.66 (33.95, 61.4)
Aviles-Santa	2016	124	233	558	14592	0.18 (0.15, 0.21)	0.98 (0.98, 0.99)	11.57 (9.43, 14.19)	0.83 (0.8, 0.86)	13.92 (11.02, 17.58)
Kim	2016	40	15	42	139	0.49 (0.38, 0.6)	0.9 (0.84, 0.94)	5.01 (2.95, 8.5)	0.57 (0.46, 0.71)	8.83 (4.44, 17.53)
Herath	2017	27	4	14	209	0.66 (0.49, 0.8)	0.98 (0.95, 0.99)	35.07 (12.96, 94.89)	0.35 (0.23, 0.53)	100.77 (30.92, 328.36)
Kenge	2017	29	4	32	728	0.48 (0.35, 0.61)	0.99 (0.99, 1)	87 (31.62, 239.4)	0.53 (0.42, 0.67)	164.94 (54.7, 497.35)
Coelho	2018	3	4	10	203	0.23 (0.05, 0.54)	0.98 (0.95, 0.99)	11.94 (2.98, 47.86)	0.78 (0.58, 1.06)	15.23 (2.99, 77.4)
Joung	2018	176	19	96	224	0.65 (0.59, 0.7)	0.92 (0.88, 0.95)	8.28 (5.33, 12.86)	0.38 (0.32, 0.45)	21.61 (12.72, 36.73)
Lopez	2018	24	6	226	857	0.1 (0.06, 0.14)	0.99 (0.98, 1)	13.81 (5.71, 33.41)	0.91 (0.87, 0.95)	15.17 (6.13, 37.55)
Nam	2018	51	3	17	318	0.75 (0.63, 0.85)	0.99 (0.97, 1)	80.25 (25.8, 249.58)	0.25 (0.17, 0.38)	318 (89.98, 1123.9)
Prakashchandra	2018	62	9	92	913	0.4 (0.32, 0.48)	0.99 (0.98, 1)	41.24 (20.94, 81.25)	0.6 (0.53, 0.69)	68.36 (32.9, 142.04)
Katulanda	2019	90	15	101	3808	0.47 (0.4, 0.54)	1 (0.99, 1)	120.1 (70.9, 203.41)	0.53 (0.46, 0.61)	226.22 (126.5, 404.53)
Cetin	2020	6	5	28	162	0.18 (0.07, 0.35)	0.97 (0.93, 0.99)	5.89 (1.91, 18.21)	0.85 (0.72, 0.99)	6.94 (1.98, 24.3)
Tucker	2020	228	122	285	6777	0.44 (0.4, 0.49)	0.98 (0.98, 0.99)	25.13 (20.56, 30.72)	0.57 (0.52, 0.61)	44.44 (34.62, 57.05)

Supplementary Material

Supplementary Table 5: Diagnostic performance of included studies assessed HbA1c 6.5% or FPG 126mg/dl for diabetic diagnosis

Author	Year	TP	FP	FN	TN	Sensitivity (95%CI)	Specificity (95%CI)	LR+ (95%CI)	LR- (95%CI)	DOR (95%CI)
Tanaka	2001	110	14	68	674	0.62 (0.54, 0.69)	0.98 (0.97, 0.99)	30.37 (17.85, 51.66)	0.39 (0.32, 0.47)	77.88 (42.34, 143.26)
lee	2013	1151	434	399	2632	0.74 (0.72, 0.76)	0.86 (0.85, 0.87)	5.25 (4.79, 5.75)	0.3 (0.28, 0.33)	17.49 (15.02, 20.38)
Aviles-Santa	2016	217	342	465	14483	0.32 (0.28, 0.36)	0.98 (0.97, 0.98)	13.79 (11.85, 16.05)	0.7 (0.66, 0.73)	19.76 (16.28, 23.98)
Herath	2017	34	38	7	175	0.83 (0.68, 0.93)	0.82 (0.76, 0.87)	4.65 (3.38, 6.4)	0.21 (0.11, 0.41)	22.37 (9.22, 54.25)
Joung	2018	213	37	59	206	0.78 (0.73, 0.83)	0.85 (0.8, 0.89)	5.14 (3.8, 6.96)	0.26 (0.2, 0.32)	20.1 (12.77, 31.63)
Lopez	2018	62	9	188	854	0.25 (0.2, 0.31)	0.99 (0.98, 1)	23.78 (11.99, 47.17)	0.76 (0.71, 0.82)	31.29 (15.28, 64.08)
Nam	2018	62	11	6	310	0.91 (0.82, 0.97)	0.97 (0.94, 0.98)	26.61 (14.82, 47.78)	0.09 (0.04, 0.2)	291.21 (103.82, 816.87)

Supplementary Table 6: Risk of bias assessment based on QUADAS- 2 domains

Author	Year	Risk of bias				Concerns of applicability		
		Patient selection	Index test	Reference standard	Flow and timing	Patient selection	Index test	Reference standard
**HbA1c 6.5%**								
Wiener	1998	Low	Low	Low	Low	Low	Low	Low
Buck	2000	Low	Low	Low	Low	High	Low	Low
Tanaka	2001	Low	Unclear	Unclear	Unclear	Low	Low	Low
Gianchadani	2011	Low	Low	Low	Low	High	Low	Yes
Hanna	2011	Low	Low	Low	Unclear	High	Low	Low
Lee	2011	Unclear	Low	Low	Unclear	Low	Low	Low
Adamu	2011	Low	Low	Low	Low	Low	Low	Low
Valentine	2011	High	Low	Low	High	Low	Low	Low
Yoon	2011	Low	Low	Low	Low	High	Low	Low
Zemlin	2011	Low	Low	Low	Low	Low	Low	Low
Homko	2012	Low	Low	Low	Low	Low	Low	Low
Marini	2012	Low	Low	Low	High	Low	Low	Low
Adamska	2012	Low	Low	Low	Low	Low	Low	Low
Bianchi	2012	Low	Low	Low	Low	Low	Low	Low
De Mulder	2012	Low	Low	High	High	High	Low	Low
Vehik	2012	Low	Low	Low	High	Low	Low	Low
kumpatla	2013	Low	Low	Low	Low	High	Low	Low
Lee	2013	Low	Low	Low	Unclear	Low	Low	Low
Alqahtani	2013	Low	Low	Low	Unclear	Low	Low	Low
Franco	2014	High	Low	Low	Low	High	Low	Low
Karnchanasorn	2016	Low	Low	Low	Low	Low	Low	Low
Kim	2016	High	Low	Low	Low	Low	Low	Low
Aviles-Santa	2016	Low	Low	Low	Unclear	Low	Low	Low
Camacho	2016	High	Low	Low	Low	Low	Low	Low
Herath	2017	Low	Low	Low	Low	Low	Low	Low
Joung	2018	Low	Low	Low	Low	Low	Low	Low

Author	Year	Risk of bias				Concerns of applicability		
		Patient selection	Index test	Reference standard	Flow and timing	Patient selection	Index test	Reference standard
Lopez	2018	Low	Low	Low	Low	Low	Low	Low
Nam	2018	Low	Low	Low	Low	Low	Low	Low
Prakashchandra	2018	Low	Low	Low	Unclear	Low	Low	Low
Coelho	2018	Low	Low	Low	Low	High	Low	Low
Aamir	2019	Low	Low	Low	High	Low	Low	Low
Thewjitcharoen	2019	Low	Low	High	High	Low	Low	Low
Basit	2020	Low	Low	Low	High	Low	Low	Low
Cetin	2020	Low	Low	Low	Low	Low	Low	Low
Tucker	2020	Low	Low	Low	Low	Low	Low	Low
**FPG 126 mg/dl**								
Lee	1997	Unclear	Low	Low	Low	Low	Low	Low
Ko	1998	Unclear	Low	Low	Low	Low	Low	Low
Nitiyanant	1998	Low	Low	Low	Low	Low	Low	Low
Chang	1998	Low	Low	Low	Low	Low	Low	Low
Croxson	1998	Low	Low	Low	Low	Low	Low	Low
Tanaka	1998	Low	Unclear	Unclear	Unclear	Low	Low	Low
Wiener	1998	Low	Low	Low	Low	Low	Low	Low
Puavilai	1999	Low	Low	Low	Low	Low	Low	Low
Shaw	1999	Low	Low	Low	Low	Low	Low	Low
Lujan	2000	Low	Low	Low	Low	Low	Low	Low
Tai	2000	Low	Low	Low	Low	Low	Low	Low
Hwu	2001	Low	Low	Low	Low	Low	Low	Low
Moran	2001	Low	Low	Low	Low	Low	Low	Low
Clark	2001	High	Low	High	High	High	Low	Low
Gatling	2001	High	Low	High	High	Low	Low	Low
Drzewoski	2001	High	Low	Low	Low	Low	Low	Low
Tanaka	2001	Low	Unclear	Unclear	Unclear	Low	Low	Low
Nakagami	2002	Low	Low	Low	Unclear	Low	Low	Low

Author	Year	Risk of bias				Concerns of applicability		
		Patient selection	Index test	Reference standard	Flow and timing	Patient selection	Index test	Reference standard
Chen	2002	High	Low	Low	Low	Low	Low	Low
Daniel	2002	Low	Low	Low	Low	High	Low	Low
Mannucci	2003	Low	Low	Low	Low	Low	Low	Low
Holt	2003	Low	Low	Low	High	High	Low	Low
Cypryk	2004	High	Low	Low	Low	Low	Low	Low
Braatvedt	2006	Low	Low	Low	Low	Low	Low	Low
Daniel	2006	Low	Unclear	Unclear	Unclear	High	Low	Low
Shrestha	2006	Low	Low	Low	Low	Low	Low	Low
Soma	2006	Low	Low	Low	Low	High	Low	Low
Al-Lawati	2007	Low	Low	Low	Low	Low	Low	Low
Gao	2008	Low	Low	Low	High	Low	Low	Low
Koike	2009	Unclear	Low	Low	High	Low	Low	Low
Hofso	2010	Low	Low	Low	High	Low	Low	Low
Bergrem	2010	Low	Low	Low	High	High	Low	Low
Tekumit	2010	Low	Low	High	High	High	Low	Low
Adamu	2011	Low	Low	Low	Low	Low	Low	Low
Yoon	2011	Low	Low	Low	Low	High	Low	Low
Homko	2012	Low	Low	Low	Low	Low	Low	Low
De Mulder	2012	Low	Low	High	High	High	Low	Low
Wen	2012	Low	Low	Low	Low	High	Low	Low
kumpatla	2013	Low	Low	Low	Low	High	Low	Low
Lee	2013	Low	Low	Low	Unclear	Low	Low	Low
Fonville	2013	Low	Low	Low	High	High	Low	Low
Huang	2015	Low	Low	Low	High	Low	Low	Low
Aekplakorn	2015	Low	Low	Low	Low	Low	Low	Low
Biswas	2015	Unclear	Low	Low	Low	Unclear	Low	Low
Kim	2016	High	Low	Low	Low	Low	Low	Low
Aviles-Santa	2016	Low	Low	Low	Unclear	Low	Low	Low

Author	Year	Risk of bias				Concerns of applicability		
		Patient selection	Index test	Reference standard	Flow and timing	Patient selection	Index test	Reference standard
Herath	2017	Low	Low	Low	Low	Low	Low	Low
Kenge	2017	High	Low	Low	High	Low	Low	Low
Joung	2018	Low	Low	Low	Low	Low	Low	Low
Lopez	2018	Low	Low	Low	Low	Low	Low	Low
Nam	2018	Low	Low	Low	Low	Low	Low	Low
Prakashchandra	2018	Low	Low	Low	Unclear	Low	Low	Low
Coelho	2018	Low	Low	Low	Low	High	Low	Low
Katulanda	2019	Low	Low	Low	High	Low	Low	Low
Cetin	2020	Low	Low	Low	Low	Low	Low	Low
Tucker	2020	Low	Low	Low	Low	Low	Low	Low
**HbA1c 6.5% or FPG 126 mg/dl**								
Tanaka	2001	Low	Unclear	Unclear	Unclear	Low	Low	Low
Lee	2013	Low	Low	Low	Unclear	Low	Low	Low
Aviles-Santa	2016	Low	Low	Low	Unclear	Low	Low	Low
Herath	2017	Low	Low	Low	Low	Low	Low	Low
Joung	2018	Low	Low	Low	Low	Low	Low	Low
Lopez	2018	Low	Low	Low	Low	Low	Low	Low
Nam	2018	Low	Low	Low	Low	Low	Low	Low

Supplementary Table 7: Pooled prevalence of diabetic melitus with reference test of OGTT 2hr ≥ 200 mg/dl

Author	Year	Country	Prevalence	95%CI
Lee	1997	Singapore	0.25	0.23, 0.28
Ko	1998	Hong Kong	0.21	0.2, 0.23
Nitiyanant	1998	Thailand	0.23	0.19, 0.27
Chang	1998	Taiwan	0.08	0.08, 0.09
Croxson	1998	UK	0.35	0.32, 0.39
Tanaka	1998	Japan	0.23	0.21, 0.25
Wiener	1998	UK	0.44	0.4, 0.49
Puavilai	1999	Thailand	0.41	0.39, 0.44
Shaw	1999	Mauritius	0.07	0.06, 0.08
Lujan	2000	Spain	0.23	0.2, 0.26
Buck	2000	Germany	0.13	0.08, 0.21
Tai	2000	Singapore	0.05	0.05, 0.06
Hwu	2001	Taiwan	0.52	0.46, 0.58
Moran	2001	Mexico	0.09	0.07, 0.11
Clark	2001	USA	0.5	0.43, 0.56
Gatling	2001	UK	0.32	0.3, 0.34
Drzewoski	2001	Poland	0.16	0.14, 0.18
Tanaka	2001	Japan	0.21	0.18, 0.23
Nakagami	2002	Multiple countries	0.06	0.06, 0.06
Chen	2002	Singapore	0.42	0.38, 0.46
Daniel	2002	Australia	0.15	0.14, 0.16
Mannucci	2003	Italy	0.24	0.2, 0.29
Holt	2003	US	0.01	0, 0.04
Cypryk	2004	Poland	0.05	0.02, 0.09
Braatvedt	2006	New Zealand	0.23	0.19, 0.28
Daniel	2006	Australia	0.12	0.11, 0.13
Shrestha	2006	Nepal	0.09	0.07, 0.11
Soma	2006	South Africa	0.12	0.07, 0.19

Author	Year	Country	Prevalence	95%CI
Al-Lawati	2007	Oman	0.1	0.09, 0.11
Gao	2008	China	0.07	0.06, 0.08
Koike	2009	Japan	0.08	0.05, 0.14
Hofso	2010	Norway	0.06	0.05, 0.09
Bergrem	2010	Norway	0.08	0.06, 0.09
Tekumit	2010	Turkey	0.54	0.45, 0.63
Gianchadani	2011	US	0.03	0.01, 0.09
Hanna	2011	UK	0.21	0.16, 0.27
Lee	2011	US	0.05	0.04, 0.06
Adamu	2011	Nigeria	0.26	0.14, 0.43
Valentine	2011	Australia	0.09	0.04, 0.22
Zemlin	2011	South Africa	0.18	0.15, 0.21
Homko	2012	America	0.15	0.11, 0.21
Marini	2012	Italy	0.13	0.11, 0.15
Adamska	2012	Poland	0.08	0.06, 0.11
Bianchi	2012	Italy	0.46	0.42, 0.49
De Mulder	2012	Netherlands	0.35	0.27, 0.44
Vehik	2012	Multiple countries	0.13	0.12, 0.15
Wen	2012	China	0.25	0.22, 0.27
Kumpatla	2013	India	0.11	0.09, 0.13
Lee	2013	Korea	0.34	0.32, 0.35
Alqahtani	2013	Saudi Arabia	0.27	0.25, 0.29
Fonvill	2013	Netherlands	0.23	0.2, 0.26
Franco	2014	Brazil	0.2	0.17, 0.24
Huang	2015	US	0.07	0.06, 0.07
Aekplakorn	2015	Thailand	0.11	0.1, 0.12
Biswas	2015	India	0.3	0.28, 0.32
Karnchanasorn	2016	US	0.07	0.06, 0.07
Kim	2016	Korea	0.35	0.29, 0.41
Aviles-Santa	2016	USA	0.04	0.04, 0.05

Author	Year	Country	Prevalence	95%CI
Camacho	2016	USA	0.04	0.02, 0.08
Herath	2017	Sri Lanka	0.16	0.12, 0.21
Kengne	2017	South Africa	0.08	0.06, 0.1
Yoon	2018	South Korea	0.17	0.12, 0.22
Joung	2018	Korea	0.53	0.48, 0.57
Lopez	2018	Colombia	0.22	0.2, 0.25
Nam	2018	Korea	0.17	0.14, 0.22
Prakaschadra	2018	South Africa	0.14	0.12, 0.17
Coelho	2018	Portugal	0.06	0.03, 0.1
Katulanda	2019	Sri Lanka	0.05	0.04, 0.05
Aamir	2019	Pakistan	0.41	0.38, 0.44
Thewjitcharoen	2019	Thailand	0.2	0.17, 0.23
Basit	2020	Pakistan	0.12	0.11, 0.13
Cetin	2020	Turkey	0.17	0.12, 0.23
Tucker	2020	USA	0.07	0.06, 0.08
Overall (I^2 = 99.16%, $p < 0.001$)			0.19	0.17, 0.20

Supplementary Table 8: Eligible studies in the systematic review and network meta-analysis (n = 73)

No.	Reference
1	Lee CH, Fook-Chong S. Evaluation of fasting plasma glucose as a screening test for diabetes mellitus in Singaporean adults. Diabet Med (1997). 14(2):119-122.
2	Ko GT, Chan JC, Yeung VT, et al. Combined use of a fasting plasma glucose concentration and HbA1c or fructosamine predicts the likelihood of having diabetes in high-risk subjects. Diabetes Care (1998). 21(8):1221-1225.
3	Nitiyanant W, Ploybutr S, Sriussadaporn S, Yamwong P, Vannasaeng S. Evaluation of the new fasting plasma glucose cutpoint of 7.0 mmol/l in detection of diabetes mellitus in the Thai population. Diabetes Res Clin Pract (1998). 41:171-6. doi: 10.1016/s0168-8227(98)00082-5.
4	Chang CJ, Wu JS, Lu FH, Lee HL, Yang YC, Wen MJ. Fasting plasma glucose in screening for diabetes in the Taiwanese population. Diabetes Care (1998). 21:1856-60. doi: 10.2337/diacare.21.11.1856.
5	Croxson S, Thomas P. Glucose tolerance test results reappraised using recent ADA criteria. Practical Diabetes International (1998). 15:178-80. doi: 10.1002/pdi.1960150608.
6	Tanaka Y, Atsumi Y, Asahina T, Hosokawa K, Matsuoka K, Kinoshita J, et al. Usefulness of revised fasting plasma glucose criterion and characteristics of the insulin response to an oral glucose load in newly diagnosed Japanese diabetic subjects. Diabetes Care (1998). 21:1133-7. doi: 10.2337/diacare.21.7.1133.
7	Wiener K, Roberts NB. The relative merits of haemoglobin A1c and fasting plasma glucose as first-line diagnostic tests for diabetes mellitus in non-pregnant subjects. Diabetic medicine : a journal of the British Diabetic Association (1998). 15:558-63. doi: 10.1002/(sici)1096-9136(199807)15:7<558::Aid-dia669>3.0.Co;2-q.
8	Puavilai G, Chanprasertyotin S, Sriphrapradaeng A. Diagnostic criteria for diabetes mellitus and other categories of glucose intolerance: 1997 criteria by the Expert Committee on the Diagnosis and Classification of Diabetes Mellitus (ADA), 1998 WHO consultation criteria, and 1985 WHO criteria. World Health Organization. Diabetes Res Clin Pract (1999). 44:21-6. doi: 10.1016/s0168-8227(99)00008-x.

9	Shaw JE, Zimmet PZ, de Courten M, Dowse GK, Chitson P, Gareeboo H, et al. Impaired fasting glucose or impaired glucose tolerance. What best predicts future diabetes in Mauritius? Diabetes Care (1999). 22:399-402. doi: 10.2337/diacare.22.3.399.
10	Martín Luján F, Costa Pinel B, Donado-Mazarrón Romero A, Basora Gallisà T, Basora Gallisà J, Piñol Moreso JL. [ADA criteria undervalues the impact of diabetes in a high-risk Spanish population]. Aten Primaria (2000). 26:517-24. doi: 10.1016/s0212-6567(00)78716-6.
11	Buck C, Thon A, Wolf A, Kohne E, Holl R. Diagnostik des Diabetes mellitus bei Mukoviszidose (CF) Stellenwert von Blutzucker, HbA1c und oralem Glukosetoleranztest. Monatsschrift Kinderheilkunde (2000). 148:698-701. doi: 10.1007/s001120050623.
12	Tai ES, Lim SC, Tan BY, Chew SK, Heng D, Tan CE. Screening for diabetes mellitus--a two-step approach in individuals with impaired fasting glucose improves detection of those at risk of complications. Diabetic medicine : a journal of the British Diabetic Association (2000). 17:771-5. doi: 10.1046/j.1464-5491.2000.00382.x.
13	Hwu CM, Kwok CF, Ku BI, Lin YT, Lee YS, Hsiao LC, et al. Undiagnosed glucose intolerance encountered in clinical practice: reappraisal of the use of the oral glucose tolerance test. Zhonghua Yi Xue Za Zhi (Taipei) (2001). 64:435-42.
14	Rodríguez-Morán M, Guerrero-Romero F. Fasting plasma glucose diagnostic criterion, proposed by the American Diabetes Association, has low sensitivity for diagnoses of diabetes in Mexican population. J Diabetes Complications (2001). 15:171-3. doi: 10.1016/s1056-8727(01)00150-7.
15	Perry RC, Shankar RR, Fineberg N, McGill J, Baron AD. HbA1c measurement improves the detection of type 2 diabetes in high-risk individuals with nondiagnostic levels of fasting plasma glucose: the Early Diabetes Intervention Program (EDIP). Diabetes Care (2001). 24:465-71. doi: 10.2337/diacare.24.3.465.
16	Gatling W, Begley J. Diagnosing diabetes mellitus in clinical practice: is fasting plasma glucose a good initial test? Practical Diabetes International (2001). 18:89-93.
17	Drzewoski J, Czupryniak L. Concordance between fasting and 2-h post-glucose challenge criteria for the diagnosis of diabetes mellitus and glucose intolerance in high risk individuals. Diabetic medicine : a journal of the British Diabetic Association (2001). 18:29-31. doi: 10.1046/j.1464-5491.2001.00403.x.

18	Tanaka Y, Atsumi Y, Matsuoka K, Mokubo A, Asahina T, Hosokawa K, et al. Usefulness of stable HbA(1c) for supportive marker to diagnose diabetes mellitus in Japanese subjects. Diabetes Res Clin Pract (2001). 53:41-5. doi: 10.1016/s0168-8227(01)00226-1.
19	Nakagami T, Qiao Q, Tuomilehto J, Balkau B, Carstensen B, Tajima N, et al. The fasting plasma glucose cut-point predicting a diabetic 2-h OGTT glucose level depends on the phenotype. Diabetes Res Clin Pract (2002). 55:35-43. doi: 10.1016/s0168-8227(01)00270-4.
20	Chen YT, Mukherjee JJ, Lee CH, Au VS, Tavintharan S. Comparing fasting plasma glucose against two-hour post-load glucose concentrations for the diagnosis of diabetes mellitus and glucose intolerance in Singaporean hospital patients. Ann Acad Med Singap (2002). 31:189-94.
21	Daniel M, Rowley KG, O'Dea K. Fasting criteria for screening: test properties and agreement with glucose tolerance. Diabetes Res Clin Pract (2002). 58:139-48. doi: 10.1016/s0168-8227(02)00133-x.
22	Mannucci E, Ognibene A, Sposato I, Brogi M, Gallori G, Bardini G, et al. Fasting plasma glucose and glycated haemoglobin in the screening of diabetes and impaired glucose tolerance. Acta Diabetol (2003). 40:181-6. doi: 10.1007/s00592-003-0109-8.
23	Holt RI, Goddard JR, Clarke P, Coleman MA. A postnatal fasting plasma glucose is useful in determining which women with gestational diabetes should undergo a postnatal oral glucose tolerance test. Diabetic medicine : a journal of the British Diabetic Association (2003). 20:594-8. doi: 10.1046/j.1464-5491.2003.00974.x.
24	Cypryk K, Czupryniak L, WilczzDski J, LewjDski A. Diabetes screening after gestational diabetes mellitus: poor performance of fasting plasma glucose. Acta Diabetologica (2004). 41:5-8.
25	Braatvedt G, Gamble G, Kyle C. Metabolic characteristics of patients with apparently normal fasting plasma glucose. N Z Med J (2006). 119:U2123.
26	Daniel M, Rowley KG, Marks E, O'Dea K. Test agreement for classifying diabetes in indigenous Australians. Aust N Z J Public Health (2006). 30:128-31. doi: 10.1111/j.1467-842x.2006.tb00104.x.
27	Shrestha UK, Singh DL, Bhattarai MD. The prevalence of hypertension and diabetes defined by fasting and 2-h plasma glucose criteria in urban Nepal. Diabetic medicine : a journal of the British Diabetic Association (2006). 23:1130-5. doi: 10.1111/j.1464-5491.2006.01953.x.

28	Soma P, Rheeder P. Unsuspected glucose abnormalities in patients with coronary artery disease. S Afr Med J (2006). 96:216-20.
29	Al-Lawati JA, Barakat MN. Fasting cut-points in determining prevalence of diabetes in an Arab population of the Middle East. Diabetes Res Clin Pract (2007). 75:241-5. doi: 10.1016/j.diabres.2006.06.018.
30	Gao W, Dong Y, Nan H, Tuomilehto J, Qiao Q. The likelihood of diabetes based on the proposed definitions for impaired fasting glucose. Diabetes Res Clin Pract (2008). 79:151-5. doi: 10.1016/j.diabres.2007.07.018.
31	Koike Y, Ogushi Y, Jin D, Sato H, Yamada T. Fasting plasma glucose reference values among young Japanese women requiring 75g oral glucose tolerance tests. Tokai J Exp Clin Med (2009). 34:15-20.
32	Hofsø D, Jenssen T, Hager H, Røislien J, Hjelmesaeth J. Fasting plasma glucose in the screening for type 2 diabetes in morbidly obese subjects. Obes Surg (2010). 20:302-7. doi: 10.1007/s11695-009-0022-5.
33	Bergrem HA, Valderhaug TG, Hartmann A, Hjelmesaeth J, Leivestad T, Bergrem H, et al. Undiagnosed diabetes in kidney transplant candidates: a case-finding strategy. Clin J Am Soc Nephrol (2010). 5:616-22. doi: 10.2215/cjn.07501009.
34	Tekumit H, Cenal AR, Polat A, Uzun K, Tataroglu C, Akinci E. Diagnostic value of hemoglobin A1c and fasting plasma glucose levels in coronary artery bypass grafting patients with undiagnosed diabetes mellitus. Ann Thorac Surg (2010). 89:1482-7. doi: 10.1016/j.athoracsur.2009.11.033.
35	Gianchandani RY, Saberi S, Zrull CA, Patil PV, Jha L, Kling-Colson SC, et al. Evaluation of hemoglobin A1c criteria to assess preoperative diabetes risk in cardiac surgery patients. Diabetes Technol Ther (2011). 13:1249-54. doi: 10.1089/dia.2011.0074.
36	Hanna FW, Geen J, Issa BG, Tahrani AA, Fryer AA. Limitations of glycosylated haemoglobin (HbA1c) in diabetes screening. Practical Diabetes (2012). 29.
37	Lee JM, Wu EL, Tarini B, Herman WH, Yoon E. Diagnosis of diabetes using hemoglobin A1c: should recommendations in adults be extrapolated to adolescents? J Pediatr (2011). 158:947-52.e1-3. doi: 10.1016/j.jpeds.2010.11.026.

38	Adamu AN. Comparative performance of HbA1c 6.5% for FPG ≥7.0 vs 2hr PG≥11.1 criteria for diagnosis of type 2 diabetes. Afr Health Sci (2011). 11:421-6.
39	Valentine NA, Alhawassi TM, Roberts GW, Vora PP, Stranks SN, Doogue MP. Detecting undiagnosed diabetes using glycated haemoglobin: an automated screening test in hospitalised patients. Med J Aust (2011). 194:160-4. doi: 10.5694/j.1326-5377.2011.tb02954.x.
40	Zemlin AE, Matsha TE, Hassan MS, Erasmus RT. HbA1c of 6.5% to diagnose diabetes mellitus--does it work for us?--the Bellville South Africa study. PloS one (2011). 6:e22558. doi: 10.1371/journal.pone.0022558.
41	Homko CJ, Zamora LC, Kerper MM, Mozzoli M, Kresge K, Boden G. A Single A1C >= 6.5% Accurately Identifies Type 2 Diabetes/Impaired Glucose Tolerance in African Americans. J Prim Care Community Health (2012). 3:235-8. doi: 10.1177/2150131911435526.
42	Marini MA, Succurro E, Arturi F, Ruffo MF, Andreozzi F, Sciacqua A, et al. Comparison of A1C, fasting and 2-h post-load plasma glucose criteria to diagnose diabetes in Italian Caucasians. Nutr Metab Cardiovasc Dis (2012). 22:561-6. doi: 10.1016/j.numecd.2011.04.009.
43	Adamska E, Waszczeniuk M, Gościk J, Golonko A, Wilk J, Pliszka J, et al. The usefulness of glycated hemoglobin A1c (HbA1c) for identifying dysglycemic states in individuals without previously diagnosed diabetes. Adv Med Sci (2012). 57:296-301. doi: 10.2478/v10039-012-0030-x.
44	Bianchi C, Miccoli R, Bonadonna RC, Giorgino F, Frontoni S, Faloia E, et al. Pathogenetic mechanisms and cardiovascular risk: differences between HbA(1c) and oral glucose tolerance test for the diagnosis of glucose tolerance. Diabetes Care (2012). 35:2607-12. doi: 10.2337/dc11-2504.
45	de Mulder M, Oemrawsingh RM, Stam F, Boersma E, Umans VA. Comparison of diagnostic criteria to detect undiagnosed diabetes in hyperglycaemic patients with acute coronary syndrome. Heart (2012). 98:37-41. doi: 10.1136/heartjnl-2011-300163.
46	Vehik K, Cuthbertson D, Boulware D, Beam CA, Rodriguez H, Legault L, et al. Performance of HbA1c as an early diagnostic indicator of type 1 diabetes in children and youth. Diabetes Care (2012). 35:1821-5. doi: 10.2337/dc12-0111.
47	Wen ZZ, Zhang XM, Mai Z, Geng DF, Wang JF. Predictive value of first fasting plasma glucose compared with admission plasma glucose for undiagnosed diabetes in a stable

	cardiology population. Clin Biochem (2012). 45:1057-63. doi: 10.1016/j.clinbiochem.2012.05.020.
48	Kumpatla S, Aravindalochanan V, Rajan R, Viswanathan V, Kapur A. Evaluation of performance of A1c and FPG tests for screening newly diagnosed diabetes defined by an OGTT among tuberculosis patients-a study from India. Diabetes Res Clin Pract (2013). 102:60-4. doi: 10.1016/j.diabres.2013.08.007.
49	Lee H, Oh JY, Sung YA, Kim DJ, Kim SH, Kim SG, et al. Optimal hemoglobin A1C Cutoff Value for Diagnosing type 2 diabetes mellitus in Korean adults. Diabetes Res Clin Pract (2013). 99:231-6. doi: 10.1016/j.diabres.2012.09.030.
50	Alqahtani N, Khan WA, Alhumaidi MH, Ahmed YA. Use of Glycated Hemoglobin in the Diagnosis of Diabetes Mellitus and Pre-diabetes and Role of Fasting Plasma Glucose, Oral Glucose Tolerance Test. Int J Prev Med (2013). 4:1025-9.
51	Fonville S, Zandbergen AA, Vermeer SE, Dippel DW, Koudstaal PJ, den Hertog HM. Prevalence of prediabetes and newly diagnosed diabetes in patients with a transient ischemic attack or stroke. Cerebrovasc Dis (2013). 36:283-9. doi: 10.1159/000353677.
52	Franco LJ, Dal Fabbro AL, Martinez EZ, Sartorelli DS, Silva AS, Soares LP, et al. Performance of glycated haemoglobin (HbA1c) as a screening test for diabetes and impaired glucose tolerance (IGT) in a high risk population--the Brazilian Xavante Indians. Diabetes Res Clin Pract (2014). 106:337-42. doi: 10.1016/j.diabres.2014.08.027.
53	Huang J, Ou HY, Karnchanasorn R, Samoa R, Chuang LM, Chiu KC, et al. Clinical implication of fasting and post-challenged plasma glucose in diagnosis of diabetes mellitus. Endocrine (2015). 48:511-8. doi: 10.1007/s12020-014-0301-3.
54	Aekplakorn W, Tantayotai V, Numsangkul S, Sripho W, Tatsato N, Burapasiriwat T, et al. Detecting Prediabetes and Diabetes: Agreement between Fasting Plasma Glucose and Oral Glucose Tolerance Test in Thai Adults. J Diabetes Res (2015). 2015:396505. doi: 10.1155/2015/396505.
55	Biswas S, Bindra M, Darotiya S, Jain dv, Agrawal V. A Retrospective Study of Fasting Plasma Glucose Cutoffs against Post Prandial Plasma Glucose. Indian Journal of Public Health Research & Development (2015). 6:199. doi: 10.5958/0976-5506.2015.00166.7.
56	Karnchanasorn R, Huang J, Ou HY, Feng W, Chuang LM, Chiu KC, et al. Comparison of the Current Diagnostic Criterion of HbA1c with Fasting and 2-Hour Plasma Glucose Concentration. J Diabetes Res (2016). 2016:6195494. doi: 10.1155/2016/6195494.

57	Kim DL, Kim SD, Kim SK, Park S, Song KH. Is an Oral Glucose Tolerance Test Still Valid for Diagnosing Diabetes Mellitus? Diabetes Metab J (2016). 40:118-28. doi: 10.4093/dmj.2016.40.2.118.
58	Avilés-Santa ML, Schneiderman N, Savage PJ, Kaplan RC, Teng Y, Pérez CM, et al. IDENTIFYING PROBABLE DIABETES MELLITUS AMONG HISPANICS/LATINOS FROM FOUR U.S. CITIES: FINDINGS FROM THE HISPANIC COMMUNITY HEALTH STUDY/STUDY OF LATINOS. Endocr Pract (2016). 22:1151-60. doi: 10.4158/ep151144.Or.
59	Camacho JE, Shah VO, Schrader R, Wong CS, Burge MR. PERFORMANCE OF A1C VERSUS OGTT FOR THE DIAGNOSIS OF PREDIABETES IN A COMMUNITY-BASED SCREENING. Endocr Pract (2016). 22:1288-95. doi: 10.4158/ep161267.Or.
60	Herath HMM, Weerarathna TP, Dahanayake MU, Weerasinghe NP. Use of HbA1c to diagnose type 2 diabetes mellitus among high risk Sri Lankan adults. Diabetes Metab Syndr (2017). 11:251-5. doi: 10.1016/j.dsx.2016.08.021.
61	Kengne AP, Erasmus RT, Levitt NS, Matsha TE. Alternative indices of glucose homeostasis as biochemical diagnostic tests for abnormal glucose tolerance in an African setting. Prim Care Diabetes (2017). 11:119-31. doi: 10.1016/j.pcd.2017.01.004.
62	Yoon JS, So CH, Lee HS, Hwang JS. Glycated hemoglobin A1c as a screening test for detecting type 2 diabetes mellitus in obese children and adolescents. J Pediatr Endocrinol Metab (2018). 31:503-6. doi: 10.1515/jpem-2017-0463.
63	Joung KH, Ju SH, Kim JM, Choung S, Lee JM, Park KS, et al. Clinical Implications of Using Post-Challenge Plasma Glucose Levels for Early Diagnosis of Type 2 Diabetes Mellitus in Older Individuals. Diabetes Metab J (2018). 42:147-54. doi: 10.4093/dmj.2018.42.2.147.
64	Lopez-Lopez J, Garay J, Wandurraga E, Camacho PA, Higuera-Escalante F, Cohen D, et al. The simultaneous assessment of glycosylated hemoglobin, fasting plasma glucose and oral glucose tolerance test does not improve the detection of type 2 diabetes mellitus in Colombian adults. PloS one (2018). 13:e0194446. doi: 10.1371/journal.pone.0194446.
65	Nam HK, Cho WK, Kim JH, Rhie YJ, Chung S, Lee KH, et al. HbA1c Cutoff for Prediabetes and Diabetes Based on Oral Glucose Tolerance Test in Obese Children and Adolescents. J Korean Med Sci (2018). 33:e93. doi: 10.3346/jkms.2018.33.e93.

66	Prakaschandra R, Naidoo DP. Fasting Plasma Glucose and the HbA1c Are Not Optimal Screening Modalities for the Diagnosis of New Diabetes in Previously Undiagnosed Asian Indian Community Participants. Ethn Dis (2018). 28:19-24. doi: 10.18865/ed.28.1.19.
67	Coelho AR, Moreira FA, Santos AC, Silva-Pinto A, Sarmento A, Carvalho D, et al. Diabetes mellitus in HIV-infected patients: fasting glucose, A1c, or oral glucose tolerance test - which method to choose for the diagnosis? BMC Infect Dis (2018). 18:309. doi: 10.1186/s12879-018-3221-7.
68	Katulanda GW, Katulanda P, Dematapitiya C, Dissanayake HA, Wijeratne S, Sheriff MHR, et al. Plasma glucose in screening for diabetes and pre-diabetes: how much is too much? Analysis of fasting plasma glucose and oral glucose tolerance test in Sri Lankans. BMC Endocr Disord (2019). 19:11. doi: 10.1186/s12902-019-0343-x.
69	Aamir AH, Ul-Haq Z, Mahar SA, Qureshi FM, Ahmad I, Jawa A, et al. Diabetes Prevalence Survey of Pakistan (DPS-PAK): prevalence of type 2 diabetes mellitus and prediabetes using HbA1c: a population-based survey from Pakistan. BMJ Open (2019). 9:e025300. doi: 10.1136/bmjopen-2018-025300.
70	Thewjitcharoen Y, Jones Elizabeth A, Butadej S, Nakasatien S, Chotwanvirat P, Wanothayaroj E, et al. Performance of HbA1c versus oral glucose tolerance test (OGTT) as a screening tool to diagnose dysglycemic status in high-risk Thai patients. BMC Endocr Disord (2019). 19:23. doi: 10.1186/s12902-019-0339-6.
71	Basit A, Fawwad A, Abdul Basit K, Waris N, Tahir B, Siddiqui IA. Glycated hemoglobin (HbA1c) as diagnostic criteria for diabetes: the optimal cut-off points values for the Pakistani population; a study from second National Diabetes Survey of Pakistan (NDSP) 2016-2017. BMJ Open Diabetes Res Care (2020). 8. doi: 10.1136/bmjdrc-2019-001058.
72	Cetin EG, Demir N, Kalkan K, Ozturkmen YA, Nazif P, Yucelen SY, et al. The Compatibility of Hemoglobin A1c with Oral Glucose Tolerance Test and Fasting Plasma Glucose. Sisli Etfal Hastan Tip Bul (2020). 54:351-6. doi: 10.14744/semb.2018.97992.
73	Tucker LA. Limited Agreement between Classifications of Diabetes and Prediabetes Resulting from the OGTT, Hemoglobin A1c, and Fasting Glucose Tests in 7412 U.S. Adults. J Clin Med (2020). 9. doi: 10.3390/jcm9072207.
